# Supplementary material for: Reaction engineering blocks ether cleavage for synthesizing chiral cyclic hemiacetals catalyzed by unspecific peroxygenase
Source: Nat Commun. 2024 Feb 9;15:1235. doi: 10.1038/s41467-024-45545-z (PMC10858125; doi:10.1038/s41467-024-45545-z)
Supplement: Supplementary file 1 — Supplenmentary Information [file 41467_2024_45545_MOESM1_ESM.pdf]

Supplementary Information

Reaction Engineering Blocks Ether Cleavage for Synthesizing Chiral Cyclic Hemiacetals Catalyzed by Unspecific Peroxygenase

Xiaofeng Han[1,2,5], Fuqiang Chen[2,5], Huanhuan Li[2,3], Ran Ge[2], Qianqian Shen[2,3], Peigao Duan[3]*, Xiang Sheng [2,4]*, Wuyuan Zhang[2,4]*

[1] College of Chemistry and Materials Science, Inner Mongolia Minzu University, Tongliao 028000, China

[2] Tianjin Institute of Industrial Biotechnology, Chinese Academy of Sciences, 32 West 7th Avenue, Tianjin 300308, China

[3] School of Chemical Engineering and Technology, Xi'an Jiaotong University, Xi'an 710049, China

[4] National Center of Technology Innovation for Synthetic Biology, 32 West 7th Avenue, Tianjin 300308, China

[5] These authors contributed equally.

Supplementary Table of Contents

1. Supplementary Methods 3

1.1 General Information 3

1.2 Preparation and purification of peroxygenases 4

1.3 Preparation of alcohol dehydrogenases 5

1.4 Analytical scale: hemiacetal synthesis reactions for GC analysis 5

1.5 Analytical scale: hemiacetal synthesis reactions for HPLC analysis 5

1.6 Reduction of the C=O double bond of lactone with ADH for reference compounds 5

1.7 Preparative scale: hemiacetal synthesis reactions for GC and NMR analysis 6

1.8 Typical procedure for chemically synthesised standards 6

1.9 Computational methods 6

2. Supplementary Figures. 8

2.1 Representative GC chromatogram of reaction 13

2.2 Peparative scale: hemiacetal synthesis reactions 18

2.3 Representative chiral HPLC chromatogram of reaction 23

2.4 Supplementary data for quantum chemical calculations 32

2.5 Supplementary data for molecular dynamics simulations 34

2.6 NMR spectra of chemically synthesised standards 36

3. Supplementary Tables 60

3.1 Supplementary Table 1. Enzyme concentration determined by immobilization 60

3.2 Supplementary Table 2. Noncycloethers catalyzed by immobilized r*Aae*UPO 61

3.3 Supplementary Table 3. NMR data of the obtained hemiacetal and lactone products 62

3.4 Supplementary Table 4. Details of the gas chromatograph and temperature profiles 65

3.5 Supplementary Table 5. Details of the HPLC chromatograph 67

4. Supplementary References 69

1. Supplementary Methods

1.1 General Information

All chemicals and solvents, unless otherwise stated, were purchased from commercial suppliers (J&K Chemical, Bide Pharmatech Ltd., Macklin and Energy Chemical Ltd.) and used without further purification. Anhydrous toluene and dichloromethane were purified and dried using calcium hydride and stored under nitrogen. Standard substances **2a**, **3a**, **5a**, **6a**, **6b**, **7a**, **7b**, **8a**, **9a**, **10a**, **11a**, and **12a** were synthesized according to the literature procedures[1-11]. Please note, due to the well-known instability, the hemiacetal compounds synthesized via chemical or enzymatic methods were used within 36 h upon the synthesis. The commercial resin carriers (LX 600, LX 603, LX 609, LX 700, LX 703, LX 704 and LX 1000) were purchased from Macklin, Mreda and Sunresin New Materials Co. Ltd. and used as received. Gas Chromatography (GC) was recorded on a GC2010 Pro from SHIMADZU. The column employed was the SH-Rtx-1 column (30 m × 0.25 mm × 0.25 µm). The temperature profile is shown on Supplementary Table 4. Data was analyzed using the software LabSolutions™ post-run analysis from SHIMADZU. High Performance Liquid Chromatography (HPLC) analyses were performed using a LC-2010C *HT* instrument from SHIMADZU. Nuclear Magnetic Resonance (NMR) spectra were recorded on a Bruker AVANCE III 400 spectrometer (Bruker Bio spin, Germany) at 400 MHz (^{1}H NMR) and 101 MHz (^{13}C NMR). NMR spectra were recorded in deuterated chloroform ($CDCl_3$) or deuterated dimethyl sulfoxide (DMSO-d_6) as a solvent, and tetramethylsilane (TMS) was used as the inert standard and Chemical shifts were reported in ppm. Abbreviations for signal coupling are as follows: singlet (s); doublet (d); triplet (t); quartet (q); multiplet (m). Coupling constants in hertz (Hz) were taken from the spectra directly and are uncorrected.

1.2 Preparation and purification of peroxygenases

The expression and preparation of recombinant unspecific peroxygenase from *Agrocybe aegerita* (r*Aae*UPO, PaDa-I) was overproduced in *P. pastoris* and purified according to a previously reported procedure[12]. After the fermentation process, the heterogeneous expression of r*Aae*UPO (PaDa-I) in *Pichia pastoris* was separated by centrifugation at 10956 × g at 4 °C for 1 h. The culture supernatant containing r*Aae*UPO which was filtered (0.22 µm) and concentrated with tangential flow filtration system (10 kDa cut-off). Upon completion, the supernatant was kept in the freezer at -80 °C for further use. The r*Aae*UPO activity after the purification was determined by using a 2,2'-azino-bis(3-ethylbenzothiazoline-6-sulfonic acid) (ABTS) assay in PBS buffer (pH 5.0, 50 mM).

DNA of r*Aae*UPO

GAACCAGGATTGCCACCAGGACCATTGGAAAATTCTTCTGCTAAATTGGTTAATGATGAAGCTCATCCATGGAAAC
CATTGAGACCAGGAGATATTAGAGGACCATGTCCAGGATTGAATACTTTGGCTTCTCATGGATATTTGCCAAGAAA
TGGAGTTGCTACTCCAGCTCAAATTATTAATGCTGTTCAAGAAGGATTTAATTTTGATAATCAAGCTGCTATTTTTGC
TACTTATGCTGCTCATTTGGTTGATGGAAATTTGATTACTGATTTGTTGTCTATTGGAAGAAAAACTAGATTGACTG
GACCAGATCCACCACCACCAGCTTCTGTTGGAGGATTGAATGAACATGGAACTTTTGAAGGAGATGCTTCTATGA
CGAGAGGAGATGCTTTTTTTGGAAATAATCATGACTTTAACGAAACGCTCTTTGAACAATTGGTTGATTATTCTAAT
AGATTTGGAGGAGGAAAATATAATTTGACTGTTGCTGGTGAGTTGAGATTTAAGAGAATACAGGATTCTATTGCTA
CTAATCCAAACTTTTCTTTCGTGGATTTCAGATTTTTTACTGCTTATGGAGAAACTACTTTTCCAGCTAATTTGTTTG
TTGATGGAAGAAGAGATGATGGACAATTGGATATGGATGCTGCTAGATCTTTTTTTCAATTTTCTAGAATGCCAGAT
GATTTTTTTAGAGCTCCATCTCCAAGATCTGGAACTGGAGTTGAAGTTGTTGTTCAAGCTCATCCAATGCAACCAG
GAAGAAATGTTGGAAAAATTAATTCTTATACTGTTGATCCAACCAGCTCTGACTTCTCTACTCCATGCCTAATGTATG
AGAAGTTTGTGAACATCACGGTTAAATCTTTGTATCCAAATCCAACTGTTCAATTGAGAAAAGCTTTGAATACTAAT
TTGGATTTTTTGTTTCAAGGAGTTGCTGCTGGATGTACTCAAGTTTTTCCATATGGAAGAGATTGA.

Immobilization of r*Aae*UPO

I. r*Aae*UPO immobilization by NH_2-resin carrier

The NH_2-resin was first washed three times with PBS buffer (pH 8, 50 mM). Then, NH_2-resin (1.0 g) was activated by glutaraldehyde in 4 mL of water (0.25 %, v/v) for 1 h at room temperature with gentle stirring (300 rpm). The residual glutaraldehyde was removed by washing three times with PBS buffer (pH 8, 50 mM). The obtained activated resin carrier (0.2 g) was then mixed with r*Aae*UPO solution at 16.67 µM and incubated for 3 h at 25 °C, 300 rpm. The immobilized r*Aae*UPO was obtained after washing three times with PBS buffer (pH 8, 50 mM), filtered, and stored at 4 °C for later use. The supernatant obtained from the procedures of filtering and washing the immobilized enzymes was collected to determine the concentration of immobilized enzyme by using a calibration curve, as shown in Supplementary Fig. 2. The amount of enzymes immobilized on the carriers and the loading efficiency are included in Supplementary Table 1.

II. r*Aae*UPO immobilization by epoxy/mesopore-resin carrier

The epoxy/mesopore-resin was first washed three times with PBS buffer (pH 8, 50 mM) and then filtered carefully to obtain the solid carriers. The obtained activated resin carrier (0.2 g) was then mixed with r*Aae*UPO solution at 16.67 µM and incubated for 3 h at 25 °C and 300 rpm. The immobilized r*Aae*UPO was obtained after washing three times with PBS buffer (pH 8, 50 mM), filtered, and stored at 4 °C for later use. The supernatant obtained from the procedures of filtering and washing the immobilized enzymes was collected to determine the concentration of immobilized enzyme. as shown in Supplementary Fig. 2 and Supplementary Table 1.

1.3 Preparation of alcohol dehydrogenases

Genes for alcohol dehydrogenase (ADH) from *Lactobacillus kefiri* DSM 20587 (GenBank: AY267012.1, *Lk*ADH)[13] was incorporated into pET24a between the restriction sites of *Nde* I and *Xho* I and expressed in *E. coli* BL21 (DE3). The cells were cultivated in 5 mL of LB medium containing 50 µg mL^{-1} kanamycin at 37 °C and 220 rpm for 6 - 8 h. Subsequently, 1% (v/v) of the seed culture was transferred into TB medium containing 50 µg mL^{-1} kanamycin at 37 °C and 220 rpm. Induction of protein expression started with the addition of 0.1 mM IPTG when the OD_{600} reached 0.6 - 0.8. Then, 0.1 mM zinc ions were added to the culture. The cultivation continued for 16 h at 20 °C and 180 rpm. The cells were harvested by centrifugation at 4 °C and 1753 × g for 10 min, washed and resuspended in PBS buffer (pH 7.4, 100 mM), and stored at 4 °C for further use.

DNA of *Lactobacillus kefiri* ADH

ATGACTGATCGTTTAAAAGGCAAAGTAGCAATTGTAACTGGCGGTACCTTGGGAATTGGCTTGGCAATCGCTGATA
AGTTTGTTGAAGAAGGCGCAAAGGTTGTTATTACCGGCCGTCACGCTGATGTAGGTGAAAAAGCTGCCAAATCAA
TCGGCGGCACAGACGTTATCCGTTTTGTCCAACACGATGCTTCTGATGAAGCCGGCTGGACTAAGTTGTTTGATAC
GACTGAAGAAGCATTTGGCCCAGTTACCACGGTTGTCAACAATGCCGGAATTGCGGTCAGCAAGAGTGTTGAAG
ATACCACAACTGAAGAATGGCGCAAGCTGCTCTCAGTTAACTTGGATGGTGTCTTCTTCGGTACCCGTCTTGGAAT
CCAACGTATGAAGAATAAAGGACTCGGAGCATCAATCATCAATATGTCATCTATCGAAGGTTTTGTTGGTGATCCAA
CTCTGGGTGCATACAACGCTTCAAAAGGTGCTGTCAGAATTATGTCTAAATCAGCTGCCTTGGATTGCGCTTTGAA
GGACTACGATGTTCGGGTTAACACTGTTCATCCAGGTTATATCAAGACACCATTGGTTGACGATCTTGAAGGGGCA
GAAGAAATGATGTCACAGCGGACCAAGACACCAATGGGTCATATCGGTGAACCTAACGATATCGCTTGGATCTGT
GTTTACCTGGCATCTGACGAATCTAAATTTGCCACTGGTGCAGAATTCGTTGTCGATGGTGGATACACTGCTCAATA
A.

1.4 Analytical scale: hemiacetal synthesis reactions for GC analysis

The cycloethers (cyclamines) (**1**-**18**) (0.5 mL) and immobilized r*Aae*UPO on LX 700 (100 mg, 1.45 µM) were added to a 1 mL reaction vial. Then, H_2O_2 from a stock solution (300 mM) was continuously injected by a syringe pump at a rate of 6 mM h^{-1} (10 µL h^{-1}) into the mixture. The reaction vial was sealed by a cap and reacted in a thermal shaker at 30 °C and 800 rpm. After the reaction, 20 µL of the reaction mixture was diluted with 180 µL of EtOAc containing dodecane as an internal standard (5 mM). The sample was dried over anhydrous Na_2SO_4 and further analyzed by gas chromatography (GC). The product concentration was obtained by using calibration curves in GC.

1.5 Analytical scale: hemiacetal synthesis reactions for HPLC analysis

After the reaction was completed, 20 µL of the reaction mixture (**1a**, **2a**, **3a**, **5a, 7a** or **12a**) was diluted with 500 µL of anhydrous dichloromethane, and then 20 µL of 2-Methylbenzoyl chloride (or acetic anhydride for **12a**) and 4 mg of DMAP were added into the solution. After incubation at 30 °C for 3 h, the organic phase was dried. The residue was redissolved in 300 µL of isopropanol and analyzed by HPLC.

1.6 Reduction of the C=O double bond of lactone with ADH for reference compounds

The lactone **8b**, isopropanol, PBS buffer (pH 7.0), *Lk*ADH, lysozyme and Dnase I were added to a 1 mL reaction vial for the asymmetric reduction of lactone. The final conditions were: [**8b**] = 5 mM, isopropanol = 5 % v/v, [PBS buffer] = 100 mM, [*Lk*ADH] = 60 µM (0.1 g mL^{-1} wet cell), [lysozyme] = 1 mg mL^{-1}, [Dnase I] = 6 U mL^{-1}, 5 h, 30 °C, 800 rpm. The reaction mixture was extracted using ethyl acetate

(extraction ratio: 1:2) and dried over Na_2SO_4. The enantiomeric excess of the samples was determined using normal phase HPLC.

1.7 Preparative scale: hemiacetal synthesis reactions for GC and NMR analysis

To a 100 mL glass bottle equipped with a magnetic stir bar (Teflon-coated) was charged with immobilized r*Aae*UPO (10 g) and substrate (50 mL), such as **1**, **2**, **5** or **10**. Then, H_2O_2 from a stock solution (300 mM) was continuously injected by a syringe pump at a rate of 6 mM h^{-1} (10 µL h^{-1}) into the solution. The reaction bottle was sealed and stirred at 30 °C, 200 rpm. After 24 h, the solution was extracted with ethyl acetate (3 times). The combined organic layers were dried over anhydrous Na_2SO_4 and the solvent removed in a vacuum. The residue was purified with flash column chromatography on silica gel (200-300 mesh) (ethyl acetate: petroleum ether = 1 : 8). Finally, 0.28, 0.69, 0.21 and 0.51 g of product (see Supplementary Figs. 19 - 27) was obtained for **1a**, **2a**, **5a** and **10a**, respectively.

1.8 Typical procedure for chemically synthesized standards

Typically, to a 50 mL oven-dried Schlenk flask containing lactone (0.5 mmol) was added 10 mL anhydrous dichloromethane under a nitrogen atmosphere. Then DIBAL-H (0.6 mmol) was added under -78 °C, the mixture was stirred 3 h until the starting material was consumed (monitored by TLC). Upon the completion of reaction. the reaction was quenched with methanol, and then Rochelle salt was added. The mixture was extracted by ethyl acetate (3 times). The combined organic layer was concentrated and purified by chromatography on silica gel (petroleum ether/ethyl acetate = 3 : 1) to afford the hemiacetal.

1.9 Computational methods

MD Simulation in THF solution

The X-ray structure of r*Aae*UPO complexed with propranolol was downloaded from the protein databank (PDB code 6ekz[14] with a resolution of 1.08 Å). According to the predicted pKa of the system by propka[15], the protonation states of each residue THF solution were determined where Asp85, ASH124, and HIP188 were set to be protonated. All missing hydrogen atoms in the system were completed using the LEAP program of amber20[16]. The force field parameters of Mg^{2+} coordinated with Cpd I and the residues and water coordinated with Mg^{2+} were fitted by "MCPB.py" [17], while the parameters of Cpd I were taken from the literature[18]. The parameters of tetrahydrofuran-2-ol, THF and non-standard residue were generated using the parmchk2 tool of AMBERTools based on the gaff2[19] force field, and the atomic charges were obtained by the RESP method[20], using HF/6-31G* level of theory in Gaussian 16 C.01 program[21]. For the standard residues of proteins, the ff19SB[22] force field is used for simulation. During the simulation process, the position of the crystal water is retained, water is described by TIP3P[23], and the protein is immersed in a rectangular THF solution box with a minimum distance of 10 angstroms between the protein and the solution boundary. To neutralize the system, counterions (Na^+ and Cl^-) were added to the surface of the protein. After system setup, the two-step minimization is used to eliminate possible conflicts in the initial structure.

First, under the constraint of using harmonic force of 15 kcal/mol/A^2 on the protein and substrate, the solvent molecules are minimized. During this process, the steepest descent method is used to optimize for 2000 steps, followed by the conjugate gradient method for another 2000 steps. After the minimization of the solvent molecules is completed, the protein constraints are released to minimize the entire system. The steepest descent method is employed again for 5000 steps, followed by the conjugate gradient method for another 5000 steps. Subsequently, the system is gradually heated under NVT ensemble, starting from 0 K and increasing to 300 K using a time step of 1 fs for a heating duration

of 100 ps. The heating process imposes restrictions on the protein and substrate with a harmonic force constant of 15 kcal /mol/A^2. Then, at a target temperature of 300 K and a pressure of 1.0 atm, the density of the system is equilibrated for 500 ps under the NPT ensemble, and a harmonic force of 5 kcal/mol/A^2 was used to constrain the position of the substrate with Cpd I in the process. The system followed by a productive MD run of 100ns.

QM Cluster Model and Methods

The quantum chemical cluster model was built based on the MD simulation results. Hierarchical clustering methods was conducted on the 10,000 frames of molecular dynamics trajectories of r*Aae*UPO in the THF solution. The structure with the highest number of conformations was selected as the initial structure to build the QM model. As shown in Supplementary Fig. 37, in order to study the process from THF to tetrahydrofuran-2-ol. We constructed Model includes residues (Cys36, Pro37, Gly38, Leu39, Phe69, Ala73, Phe76, Ala77, Arg169, Phe121, Phe188, Arg189, Thr192, Glu196, Phe199) that may interact with the substrate or porphyrin, a molecule of water, Cpd I and a molecule of THF. This model is called $\mathbf{E_{Cpd\ I}}$**:1** (Supplementary Fig. 37). $\mathbf{E_{Cpd\ I}}$**:1** contains a total of 255 atoms and has a total charge of +1. The doublet spin state and quartet spin are calculated separately. In addition, we replaced the THF in $\mathbf{E_{cpd\ I}}$**:1** with tetrahydrofuran-2-ol to construct $\mathbf{E_{Cpd\ I}}$**:1a** (Supplementary Fig. 38), which is used to calculate the process from THF to tetrahydrofuran-2-ol. $\mathbf{E_{Cpd\ I}}$**:1a** contains a total of 256 atoms and has a total charge of +1. According to the calculation results of $\mathbf{E_{Cpd\ I}}$**:1**, only the doublet spin state was considered. The truncated part of the model is saturated with hydrogen atoms. All structures are in their lowest energy conformation.

All QM calculations in this study were performed using the Gaussian 16[21] program, with B3LYP-D3(BJ) hybrid functional[24-27]. It has been established that density functional theory (DFT), in particular the B3LYP functional with the Grimme dispersion correction, offers reliable insights into the reaction mechanisms and selectivity of heme-dependent enzymes[28-31]. Therefore, B3LYP-D3(BJ) was chosen to investigate the reaction mechanism of r*Aae*UPO-catalyzed transformation in the present study. The geometry optimizations used the Def2-SV(P) basis[32]. Frequency calculations were performed at the same theoretical level as geometric optimization to obtain the zero-point energies (ZPEs). The solvation effect in tetrahydrofuran solution is obtained using the SMD model with a dielectric $\varepsilon = 4.0$[33] at the same level of theory. The single point calculation with a large basis set uses the Def2-TZV(P)[32] basis set. ZPEs and solvation effects were added to the single point energies from the large basis set calculations.

Steered MD simulations and Umbrella sampling simulations

According to the pKa predicted by propka[15], the protonation state of the protein at pH=4 and pH=9 was reset. The system setup for Umbrella sampling[34] is the same as for MD simulations, but the solvent is replaced with water. In addition, THF and tetrahydrofuran-2-ol were manually placed at the active site. Before starting steered MD simulations, the substrate was fixed at the active site and simulated for 20ns, and the simulated structure was used as the initial structure of steered MD[35], the harmonic force used is 5 kcal /mol/A^2. During the steered MD simulations, we applied pulling velocitie of 0.0003 Å /ps and a spring force constant of 50 kcal/mol/ Å^2. We use the distance between the oxygen of Cpd I and the oxygen on the ring of the substrate as the criterion. The stretch distance is pulled from 3 Å to 24 Å. Then, a snapshot is taken every 0.25 Å from the steered MD simulations trajectory and used as a window for umbrella sampling, with a total of 85 windows. Each window is sampled 5 ns after equilibrating 100ps, and harnonic restraint is 10 kcal/mol/ Å^2. Then, the data of the last 4 ns of each window sample uses WHAM[36] to calculate the mean force profile (PMF).

2. Supplementary Figures

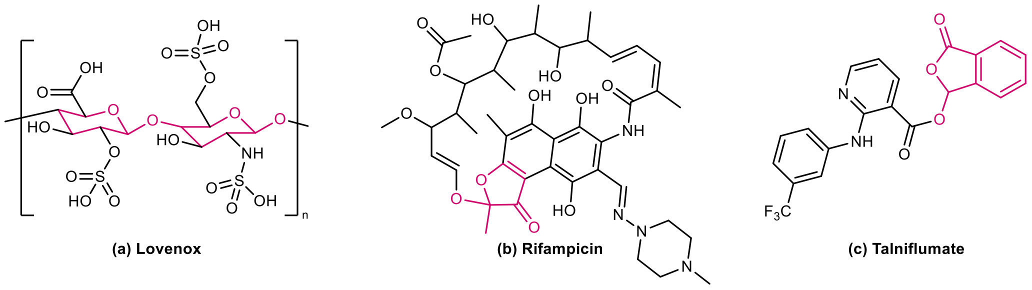

Supplementary Fig. 1. Available cyclic hemiacetal units (claret red) in drug synthesis. (a) Lovenox for antithrombotic therapy; (b) Rifampicin for broad-spectrum antibiotics and (c) Talniflumate for mucin regulator.

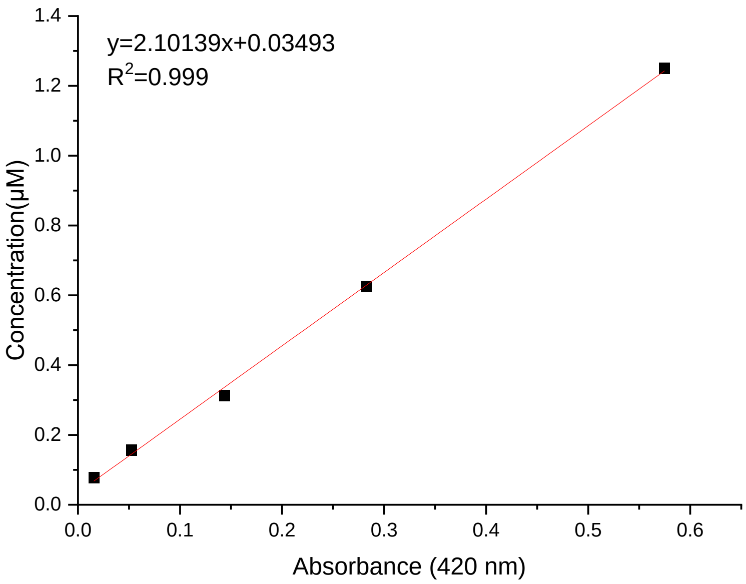

Supplementary Fig. 2. Calibration curve obtained at different r*Aae*UPO concentrations using the ABTS assay.

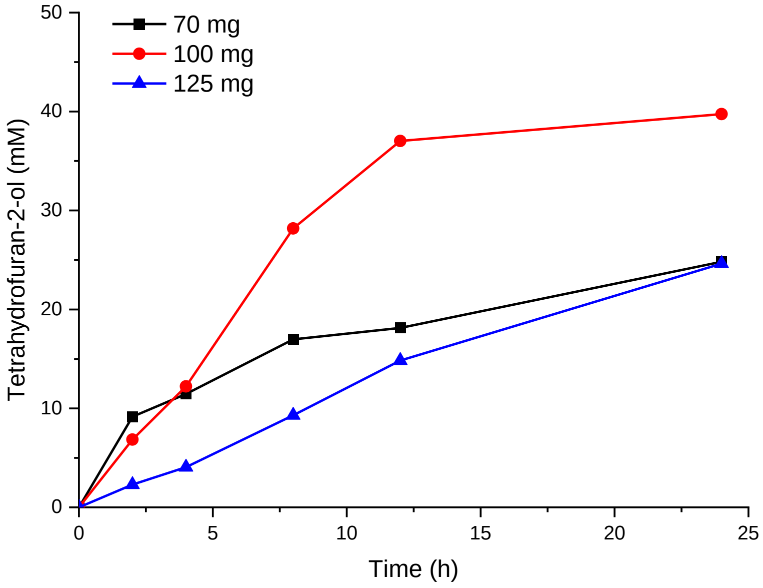

Supplementary Fig. 3. Time course of the oxyfunctionalization of THF under neat reaction conditions. Conditions: [THF] = 0.5 mL (12.3 M), [immobilized r*Aae*UPO] = 70-125 mg (corresponding to 1.02 - 1.81 µM), [H_2O_2] = 6 mM h^{-1}, 30 °C, 24 h. The reported value is based on the mean value of a duplicate experiment.

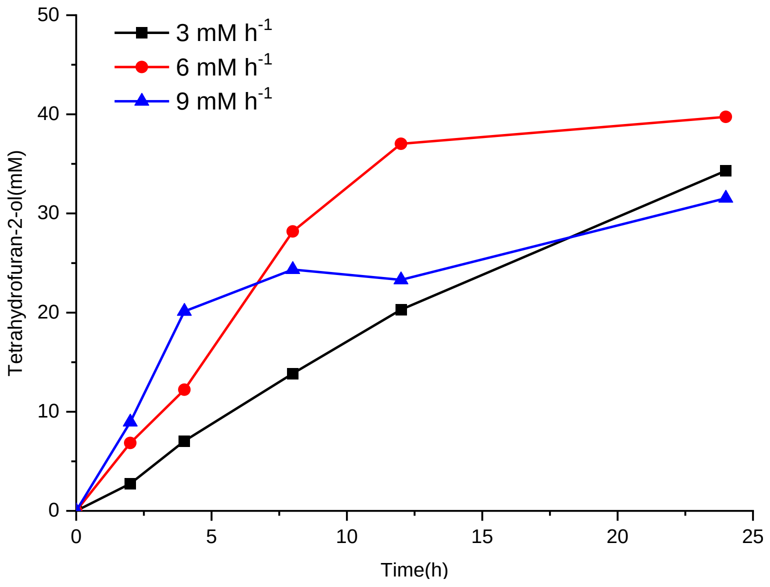

Supplementary Fig. 4. Time course of the oxyfunctionalization of THF under different concentrations of H_2O_2. Conditions: [THF] = 0.5 mL (12.3 M), [immobilized r*Aae*UPO] = 100 mg (corresponding to 1.45 µM), [H_2O_2] = 3 - 9 mM h^{-1}, 30 °C, 24 h. The reported value is based on the mean value of a duplicate experiment.

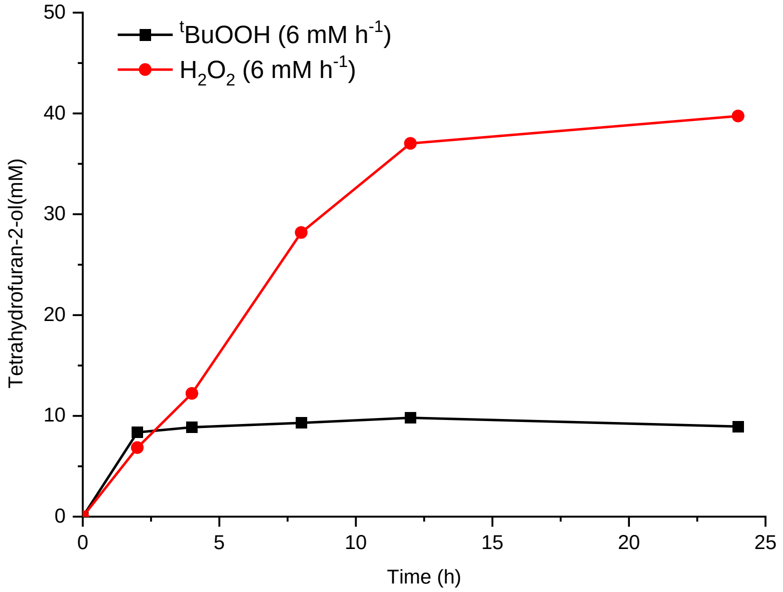

Supplementary Fig. 5. Time course of the oxyfunctionalization of THF under the same concentration of H_2O_2 and ^{t}BuOOH. Conditions: [THF] = 0.5 mL (12.3 M), [immobilized r*Aae*UPO] = 100 mg (corresponding to 1.45 μM), [H_2O_2] = 6 mM h^{-1}, [^{t}BuOOH] = 6 mM h^{-1}, 30 °C, 24 h. The reported value is based on the mean value of a duplicate experiment.

2.1 Representative GC chromatogram of reaction

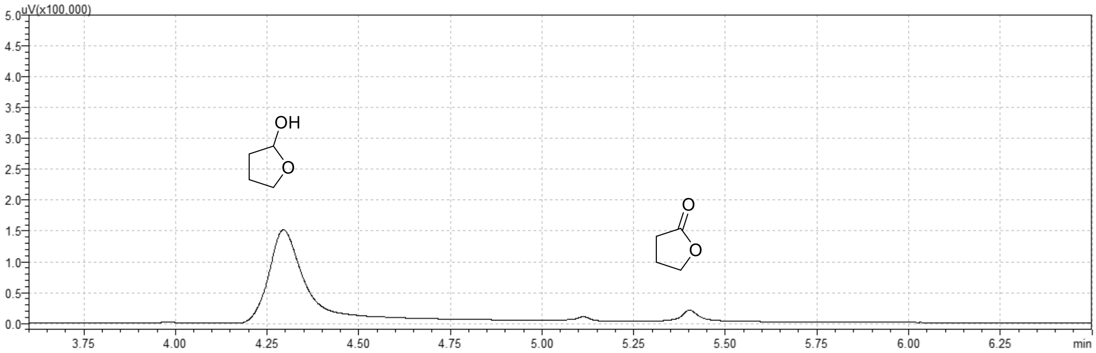

Supplementary Fig. 6. Representative GC chromatogram of reaction mixture after 24 h.

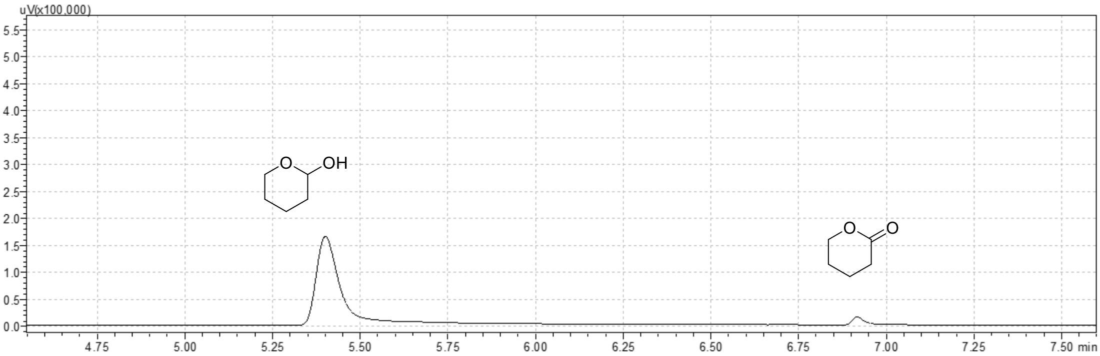

Supplementary Fig. 7. Representative GC chromatogram of reaction mixture after 24 h.

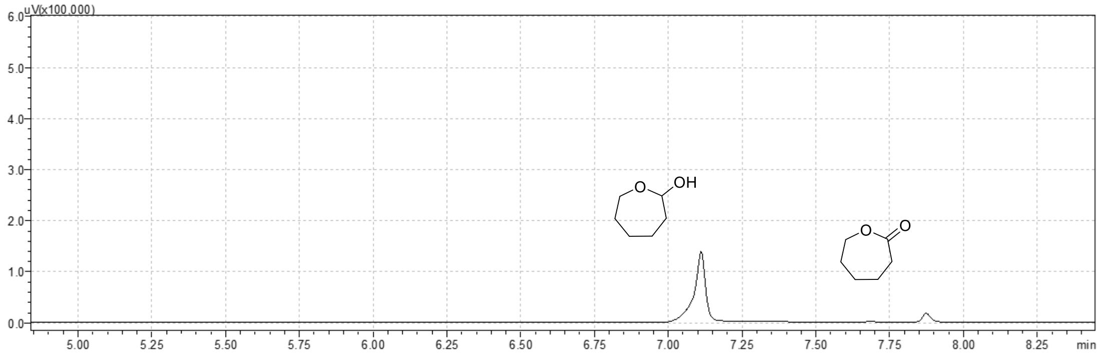

Supplementary Fig. 8. Representative GC chromatogram of reaction mixture after 24 h.

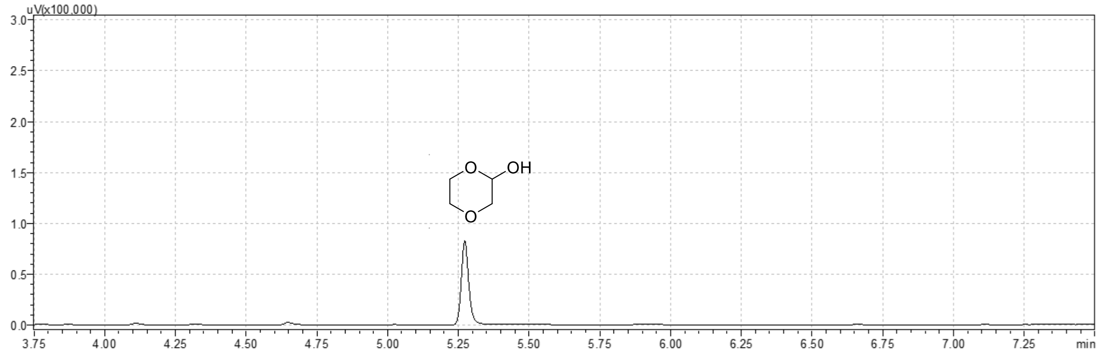

Supplementary Fig. 9. Representative GC chromatogram of reaction mixture after 24 h.

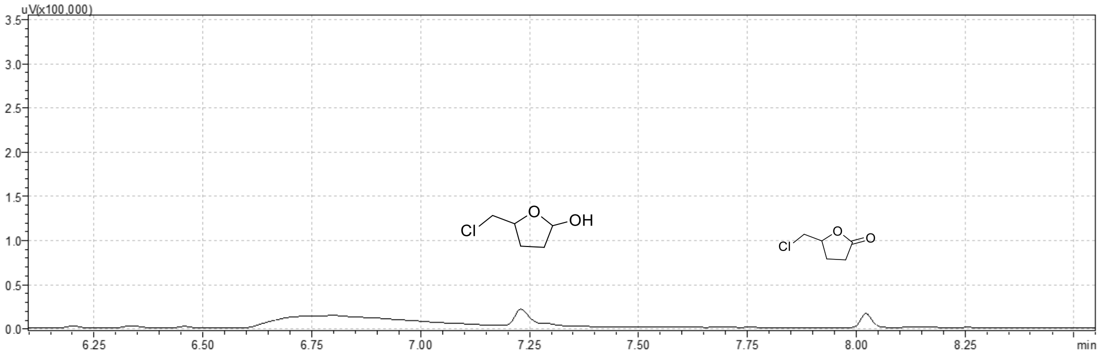

Supplementary Fig. 10. Representative GC chromatogram of reaction mixture after 24 h.

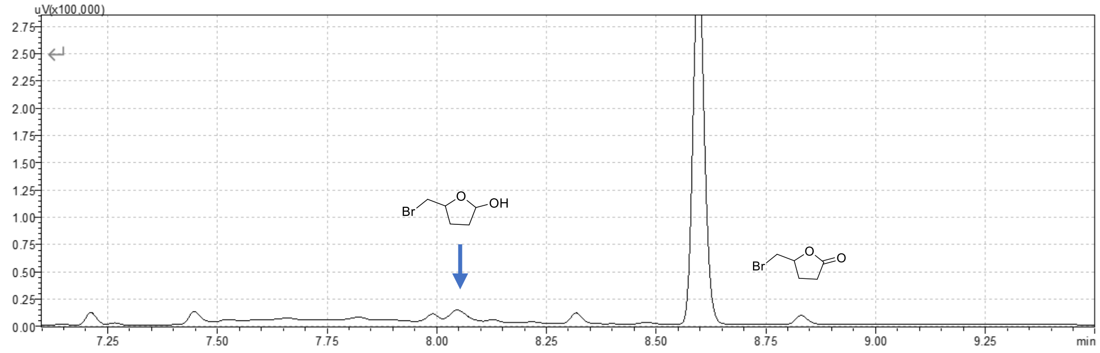

Supplementary Fig. 11. Representative GC chromatogram of reaction mixture after 24 h, the peak at 8.60 min was the internal standard, the tiny peak of impurity was derived from starting material.

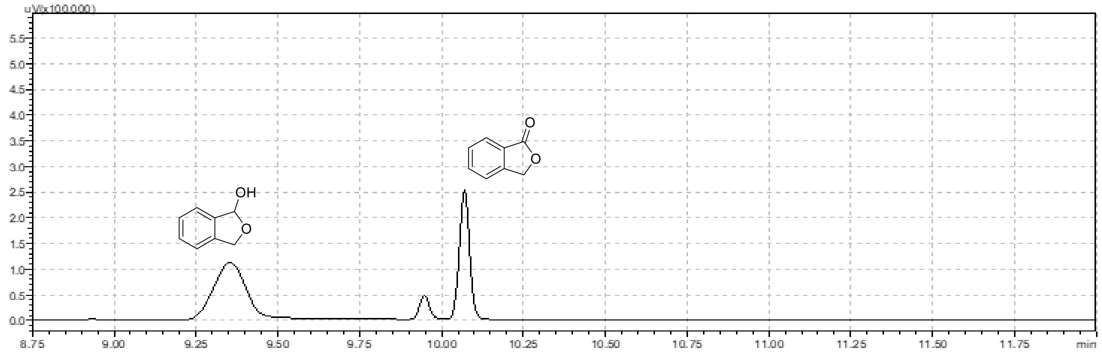

Supplementary Fig. 12. Representative GC chromatogram of reaction mixture after 24 h, the peak of impurity at 9.95 min was derived from starting material.

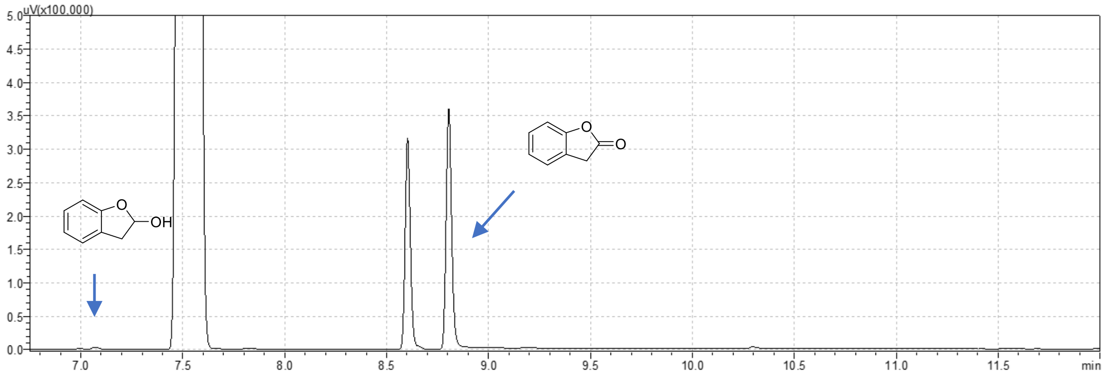

Supplementary Fig. 13. Representative GC chromatogram of reaction mixture after 24 h. the peak at 7.55 min and 8.62 min were substrate **9** and internal standard respectively.

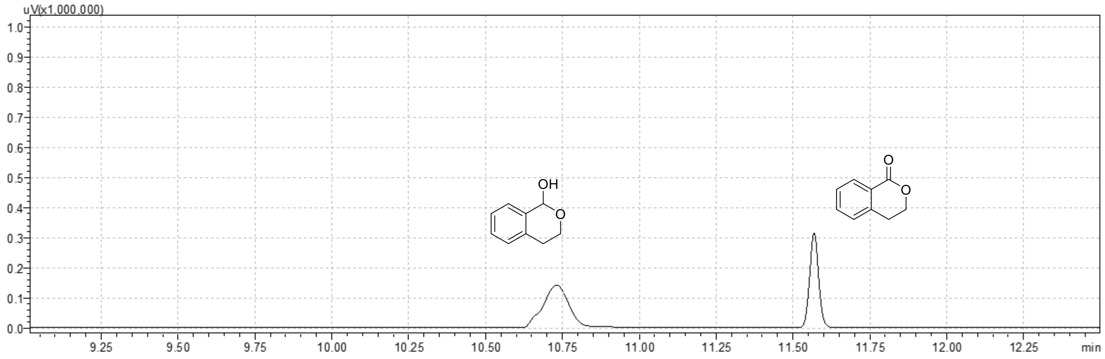

Supplementary Fig. 14. Representative GC chromatogram of reaction mixture after 24 h.

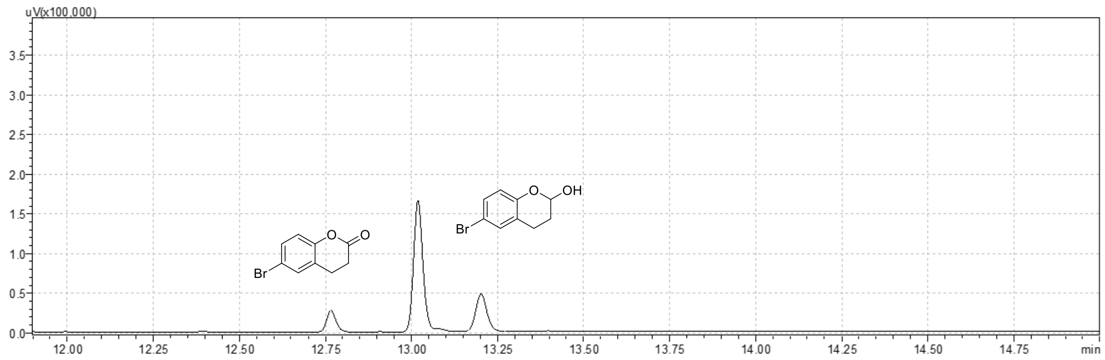

Supplementary Fig. 15. Representative GC chromatogram of reaction mixture after 24 h， the peak at 13.02 min was the internal standard.

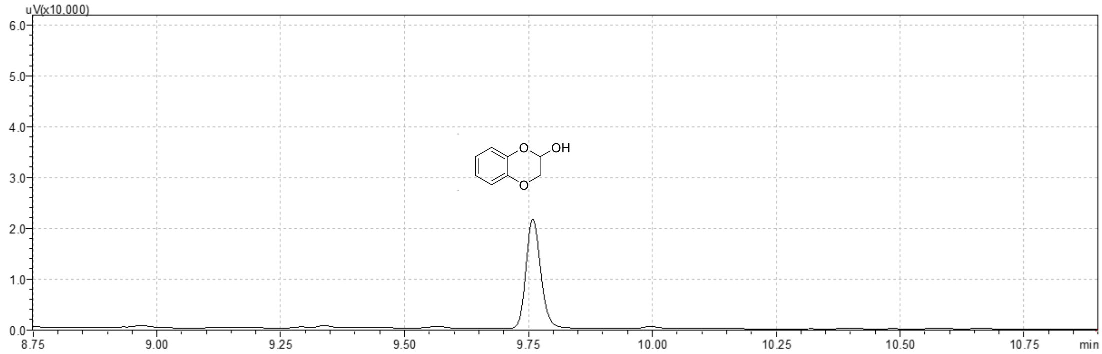

Supplementary Fig. 16. Representative GC chromatogram of reaction mixture after 24 h.

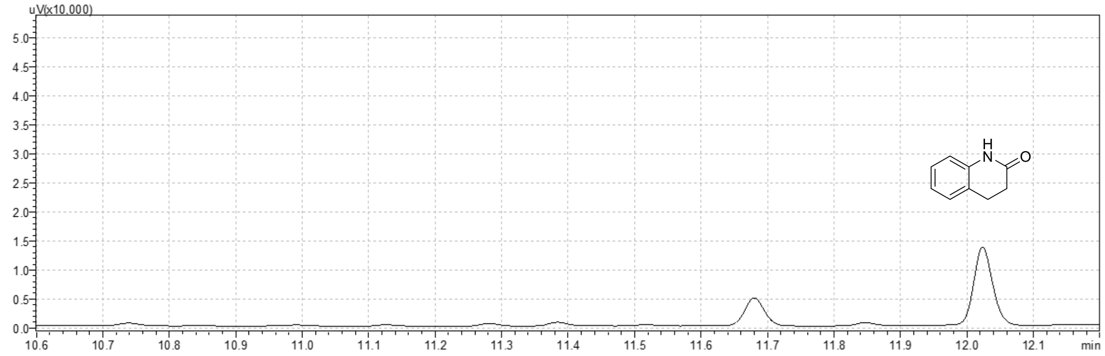

Supplementary Fig. 17. Representative GC chromatogram of reaction mixture after 24 h, the peak of impurity at 11.68 min was derived from starting material.

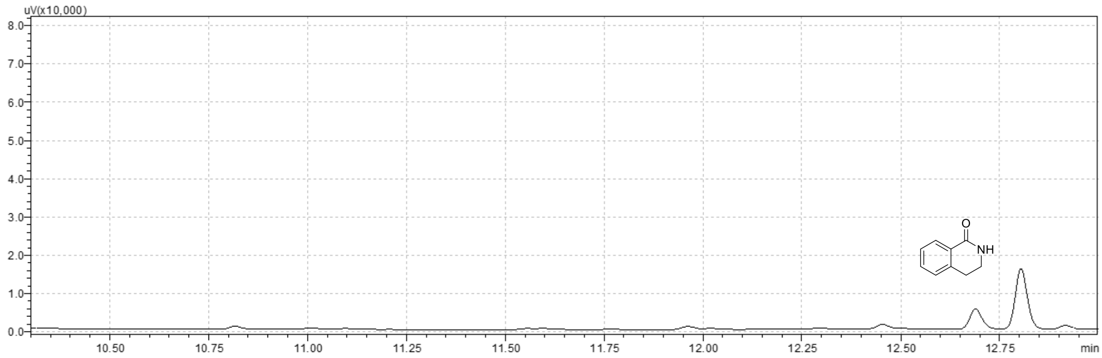

Supplementary Fig. 18. Representative GC chromatogram of reaction mixture after 24 h, the peak of impurity at 12.81 min was derived from starting material.

2.2 Peparative scale: hemiacetal synthesis reactions

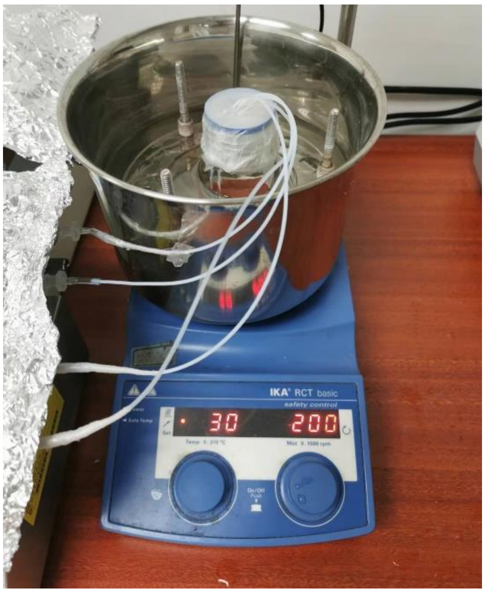

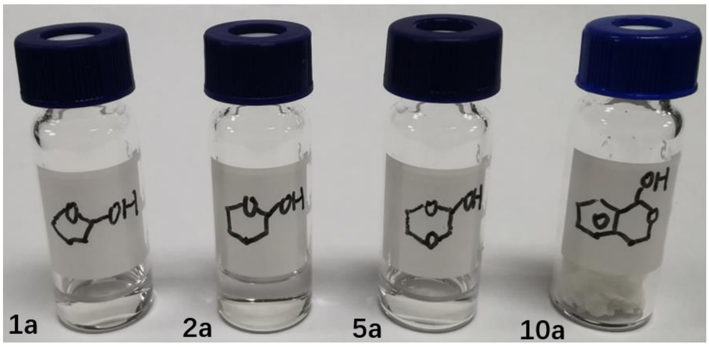

Supplementary Fig. 19. The hemiacetal synthesis and isolated products of **1a**, **2a**, **5a** and **10a**.

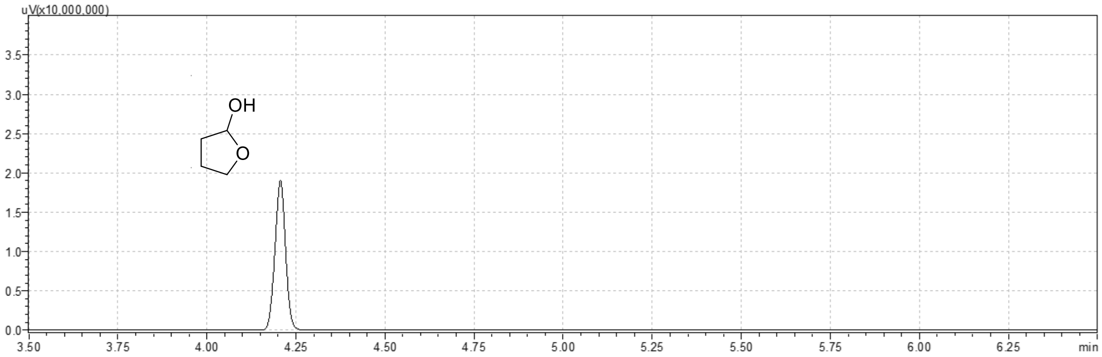

Supplementary Fig. 20. GC spectrum of **1a** obtained from preparative synthesis.

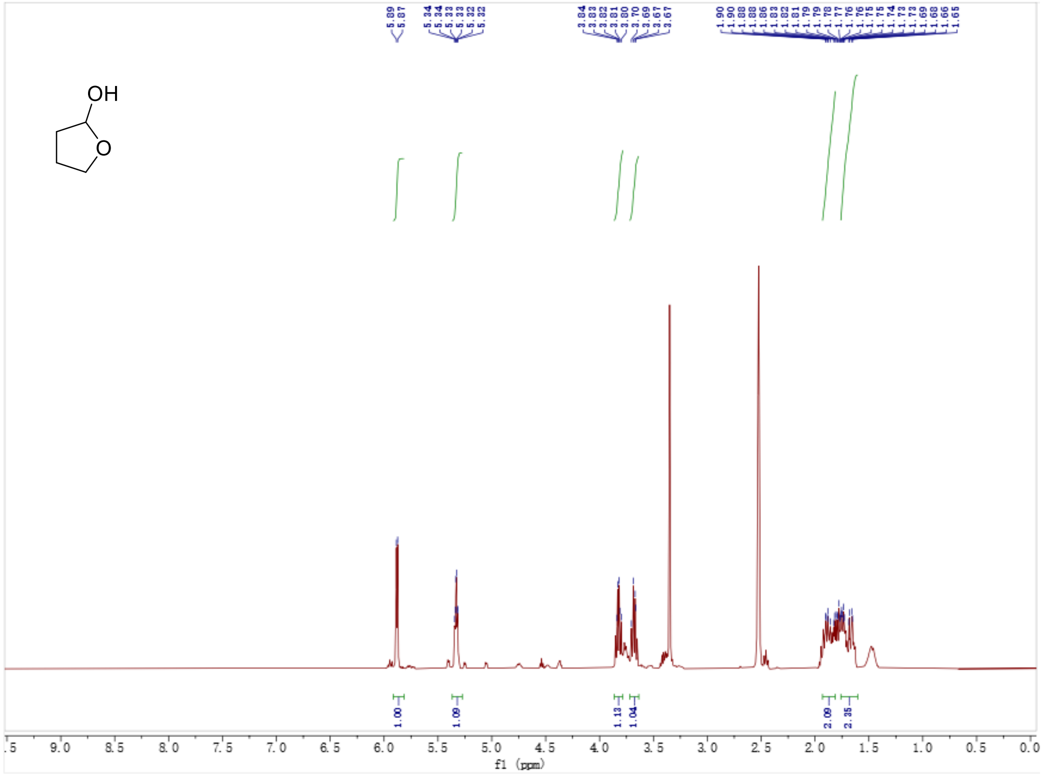

Supplementary Fig. 21. ^{1}H NMR of **1a** obtained from preparative synthesis.

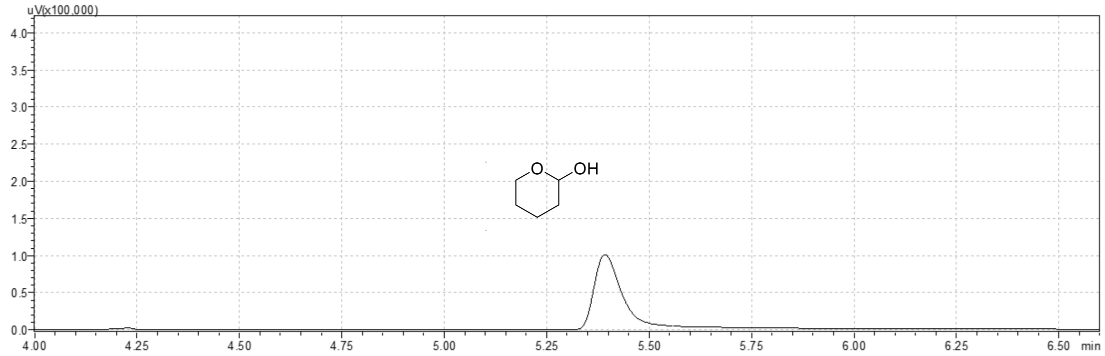

Supplementary Fig. 22. GC spectrum of **2a** obtained from preparative synthesis.

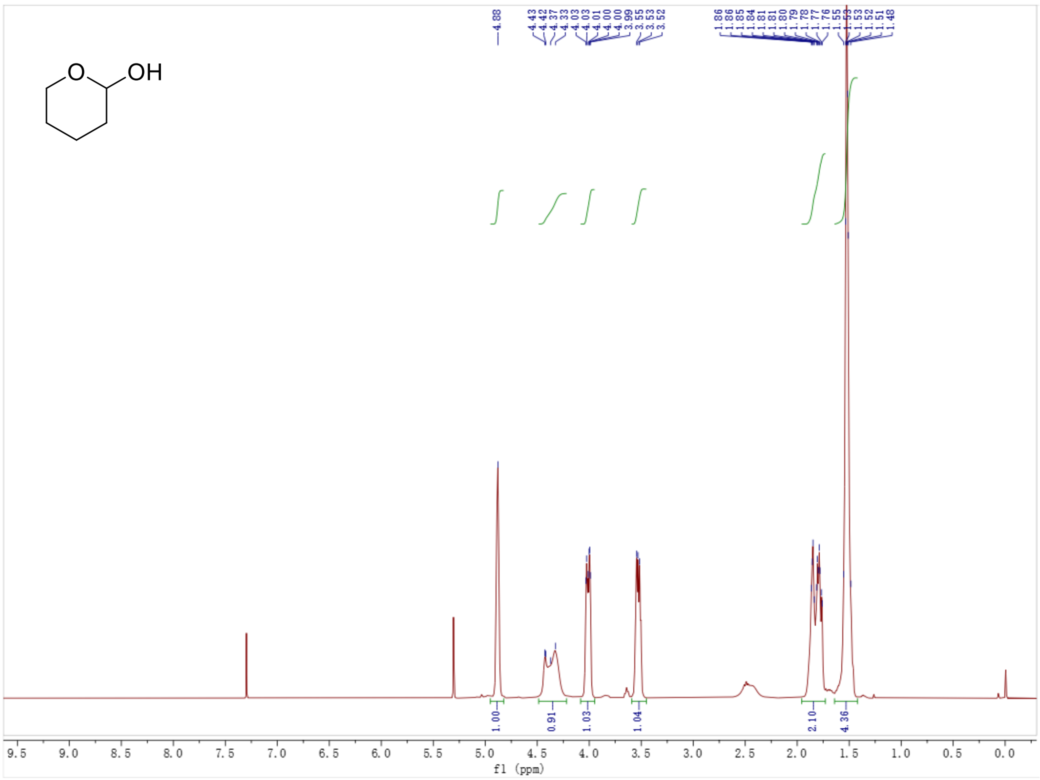

Supplementary Fig. 23. ^1H NMR of **2a** obtained from preparative synthesis.

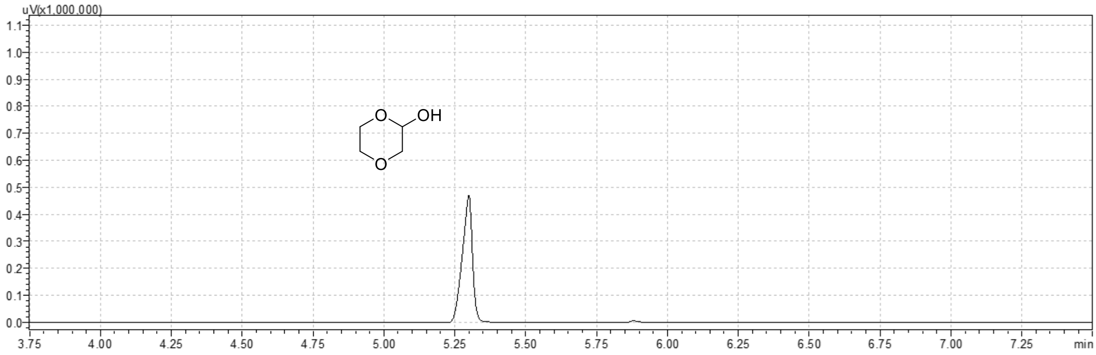

Supplementary Fig. 24. GC spectrum of **5a** obtained from preparative synthesis.

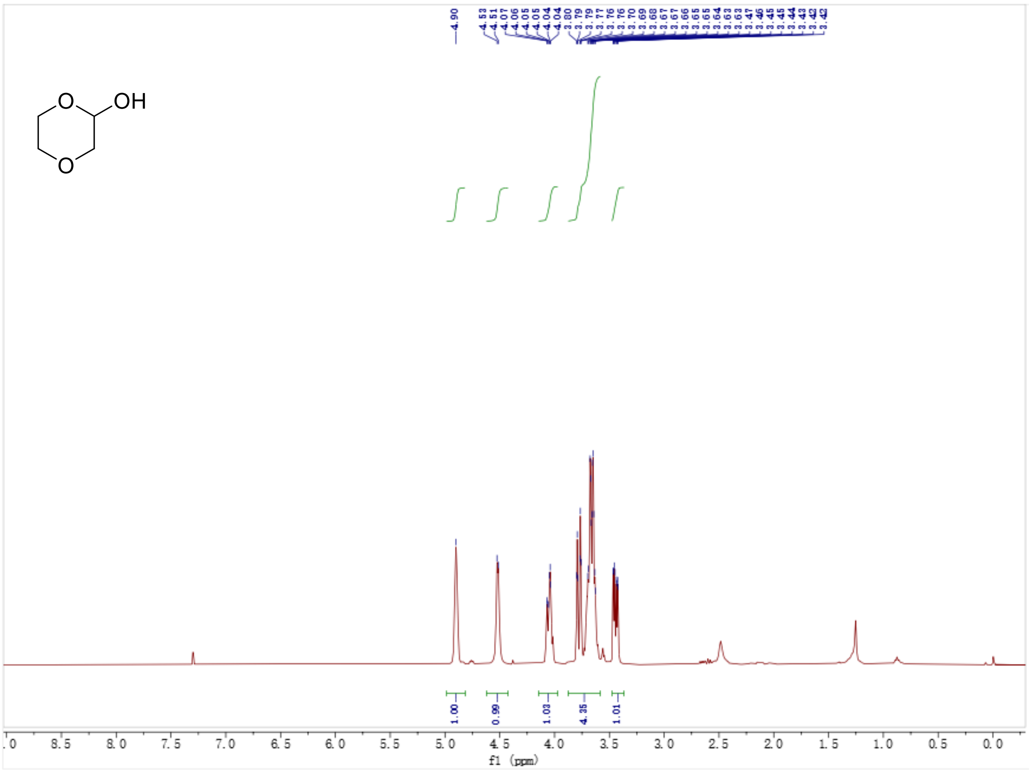

Supplementary Fig. 25. ^{1}H NMR of **5a** obtained from preparative synthesis.

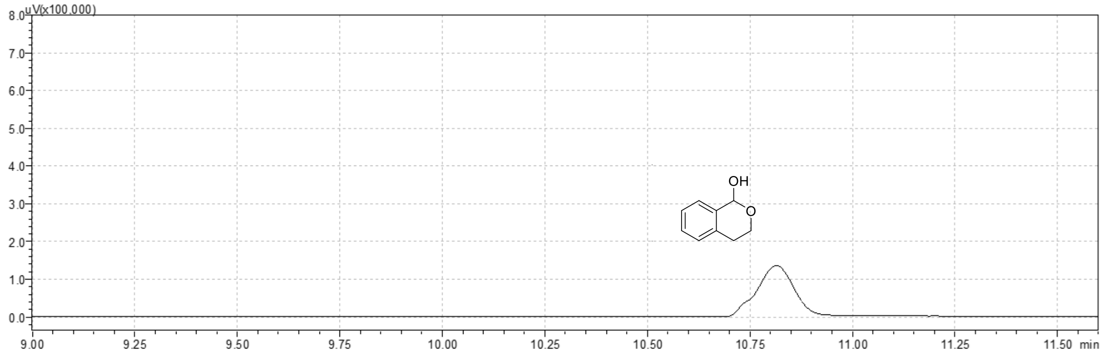

Supplementary Fig. 26. GC spectrum of **10a** obtained from preparative synthesis.

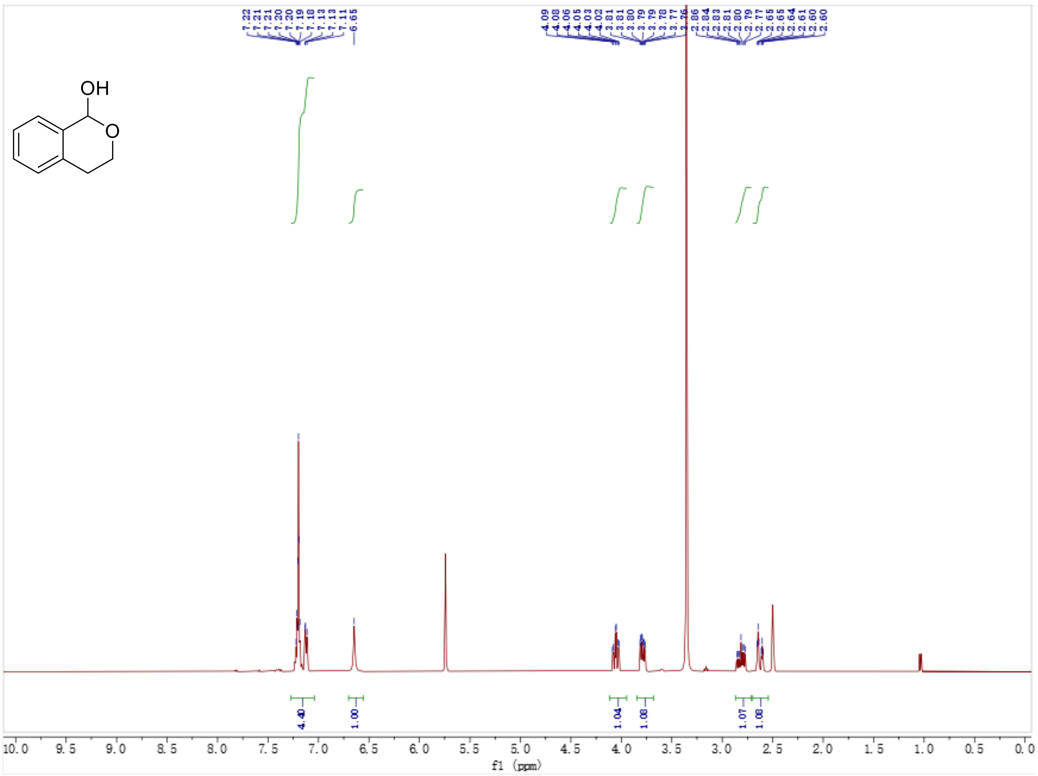

Supplementary Fig. 27. ^{1}H NMR of **10a** obtained from preparative synthesis.

2.3 Representative chiral HPLC chromatogram of reaction

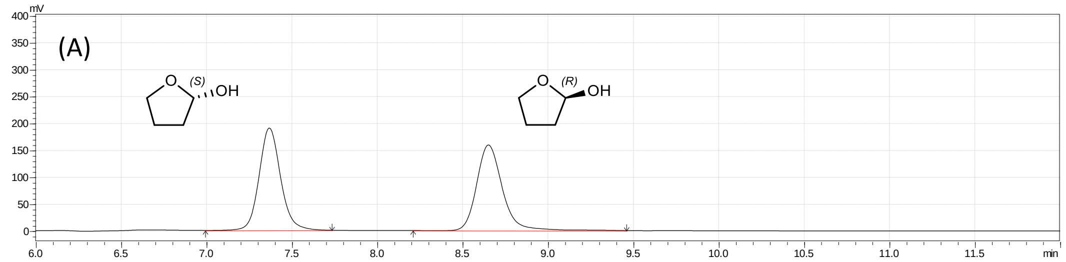

Peak#	Ret. Time [min]	Area [mV*min]	Hight [mV]	Area (%)
1	7.368	1661259	190282	50.071
2	8.859	1656521	159199	49.929

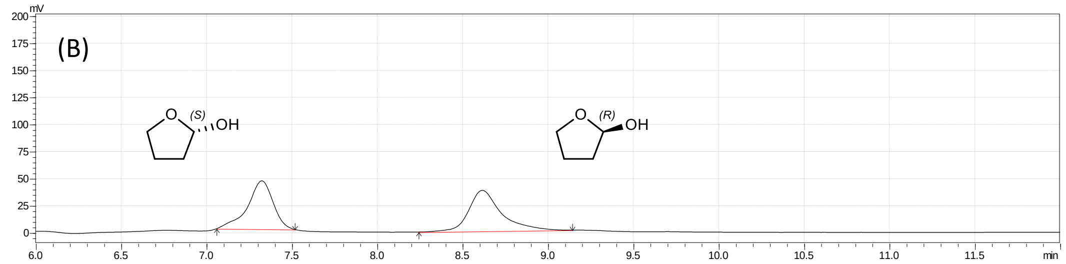

Peak#	Ret. Time [min]	Area [mV*min]	Hight [mV]	Area (%)
1	7.324	464793	44643	48.181
2	8.616	499883	37782	51.819

Supplementary Fig. 28. Representative chiral HPLC chromatogram of (A) rac-**1a** and (B) the reaction mixture after esterification (2-Methylbenzoyl chloride).

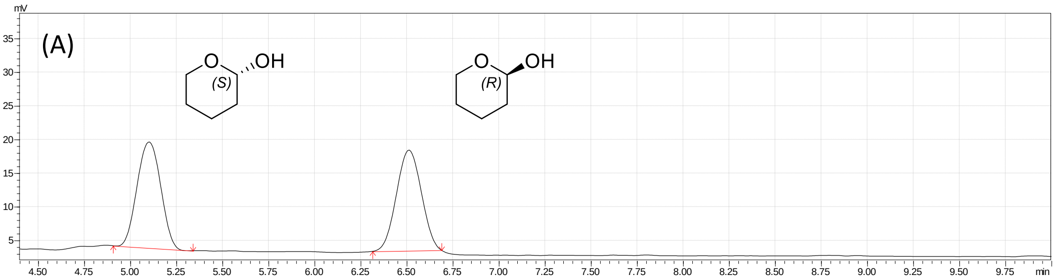

Peak#	Ret. Time [min]	Area [mV*min]	Hight [mV]	Area (%)
1	5.221	140412	15742	49.932
2	6.632	140794	14960	50.068

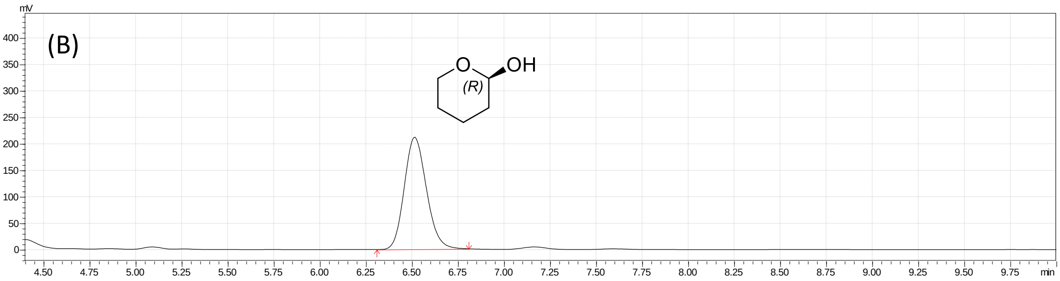

Peak#	Ret. Time [min]	Area [mV*min]	Hight [mV]	Area (%)
1	6.515	1771858	211704	100.000

Supplementary Fig. 29. Representative chiral HPLC chromatogram of (A) rac-**2a** and (B) the reaction mixture after esterification (2-Methylbenzoyl chloride).

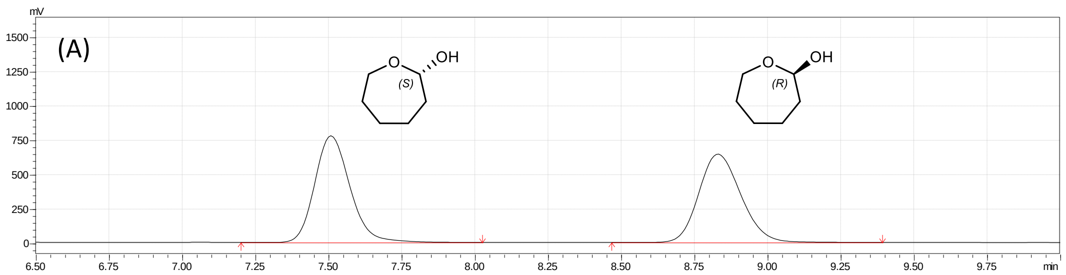

Peak#	Ret. Time [min]	Area [mV*min]	Hight [mV]	Area (%)
1	7.508	6478447	775822	50.279
2	8.831	6406614	642472	49.721

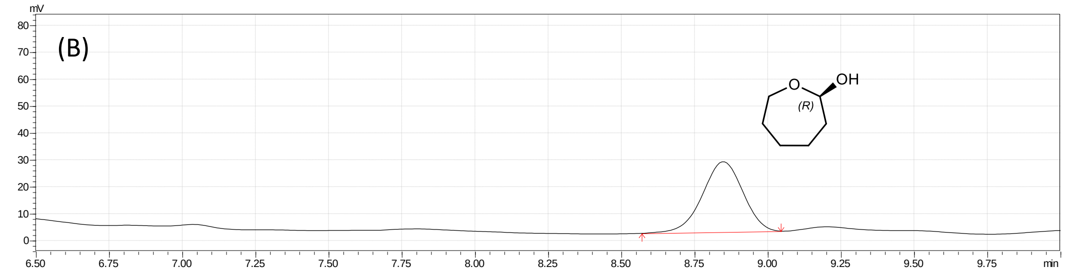

Peak#	Ret. Time [min]	Area [mV*min]	Hight [mV]	Area (%)
1	8.847	101533	10694	100.000

Supplementary Fig. 30. Representative chiral HPLC chromatogram of (A) rac-**3a** and (B) the reaction mixture after esterification (2-Methylbenzoyl chloride).

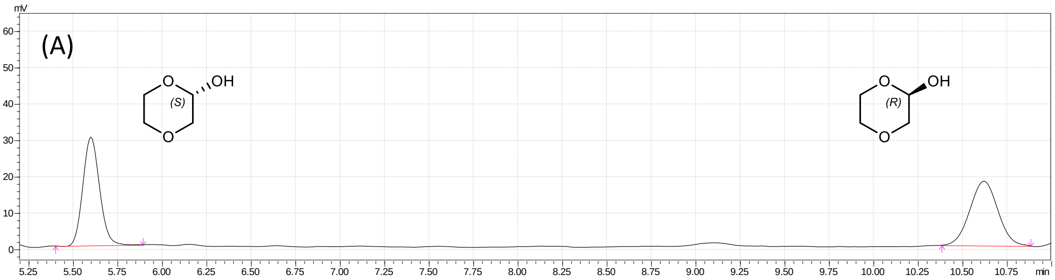

Peak#	Ret. Time [min]	Area [mV*min]	Hight [mV]	Area (%)
1	5.600	193189	29811	49.952
2	10.621	193563	17685	50.048

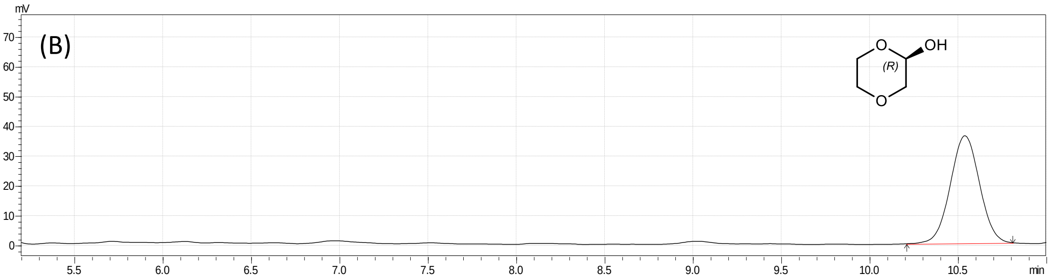

Peak#	Ret. Time [min]	Area [mV*min]	Hight [mV]	Area (%)
1	10.539	415350	36090	100.000

Supplementary Fig. 31. Representative chiral HPLC chromatogram of (A) rac-**5a** and (B) the reaction mixture after esterification (2-Methylbenzoyl chloride).

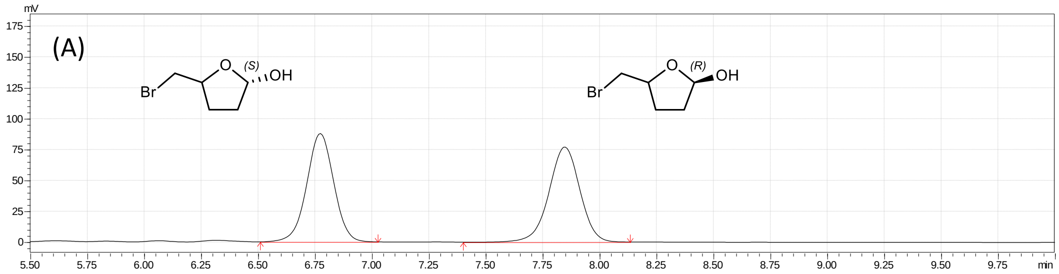

Peak#	Ret. Time [min]	Area [mV*min]	Hight [mV]	Area (%)
1	6.774	727002	87791	49.955
2	7.847	728318	76998	50.045

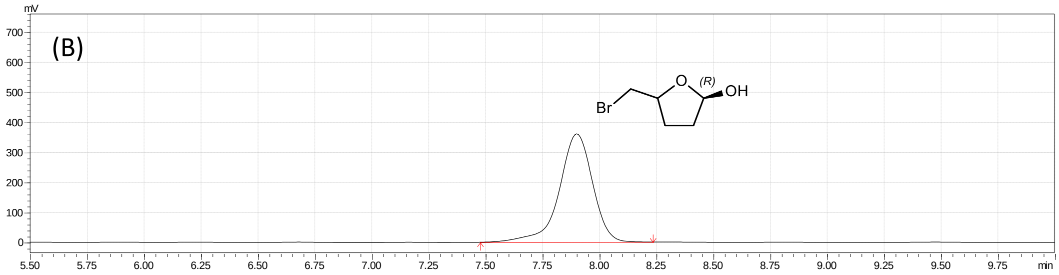

Peak#	Ret. Time [min]	Area [mV*min]	Hight [mV]	Area (%)
1	7.900	3661538	360783	100.000

Supplementary Fig. 32. Representative chiral HPLC chromatogram of (A) rac-**7a** and (B) the reaction mixture after esterification (2-Methylbenzoyl chloride).

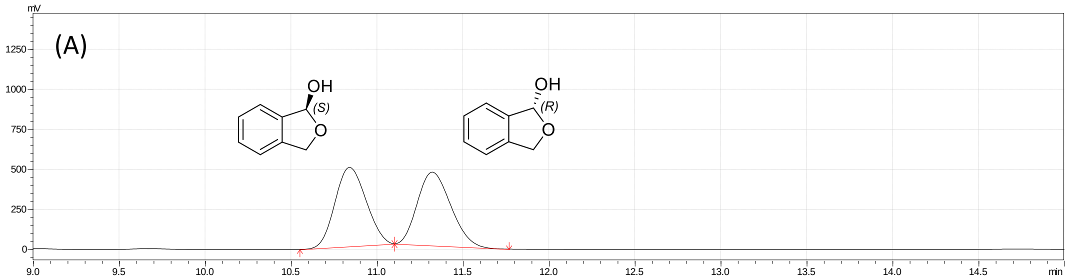

Peak#	Ret. Time [min]	Area [mV*min]	Hight [mV]	Area (%)
1	10.841	6211156	494326	50.125
2	11.322	6180140	458812	49.875

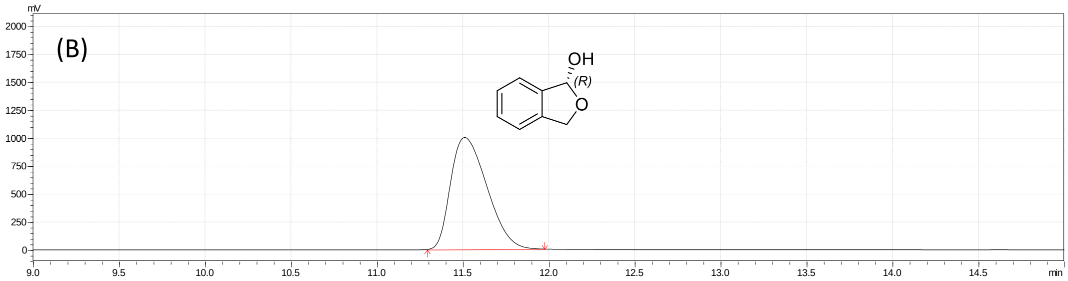

Peak#	Ret. Time [min]	Area [mV*min]	Hight [mV]	Area (%)
1	11.511	14705517	999391	100.000

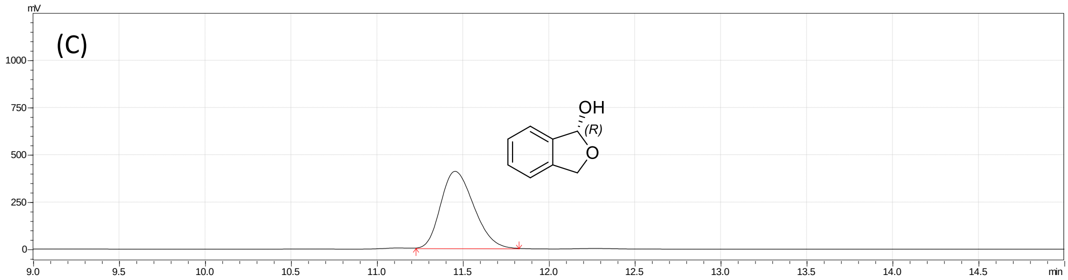

Peak#	Ret. Time [min]	Area [mV*min]	Hight [mV]	Area (%)
1	11.455	5423098	406413	100.000

Supplementary Fig. 33. Representative chiral HPLC chromatogram of (A) rac-**8a**, (B) *R*-**8a** and (C) the reaction mixture after 24 h.

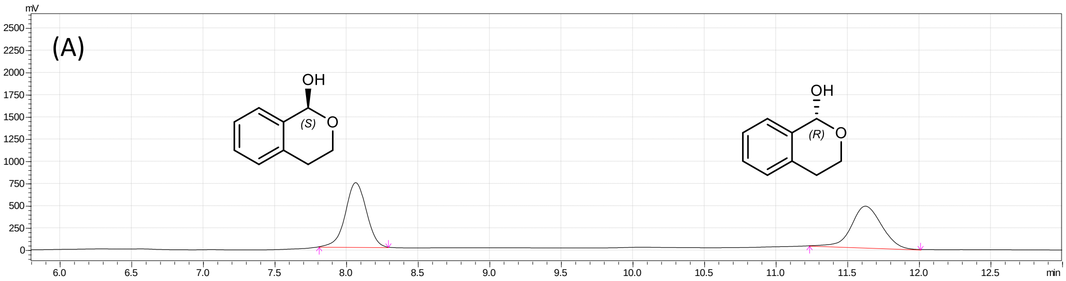

Peak#	Ret. Time [min]	Area [mV*min]	Hight [mV]	Area (%)
1	8.075	523568	38536	49.984
2	11.601	523910	18646	50.016

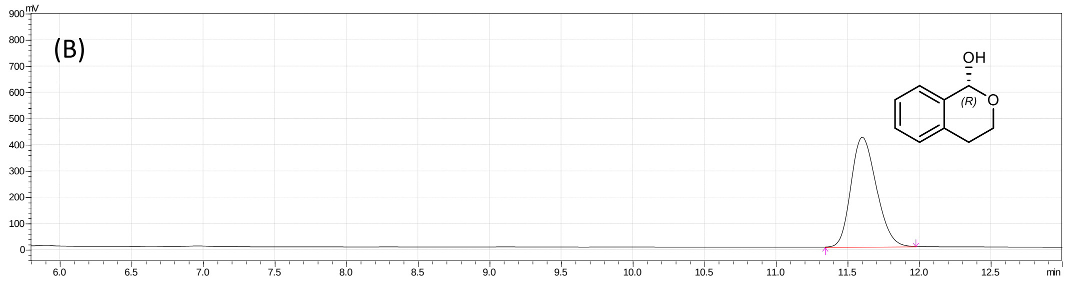

Peak#	Ret. Time [min]	Area [mV*min]	Hight [mV]	Area (%)
1	11.603	5141013	417741	100.000

Supplementary Fig. 34. Representative chiral HPLC chromatogram of (A) rac-**10a** and (B) reaction mixture after 24 h.

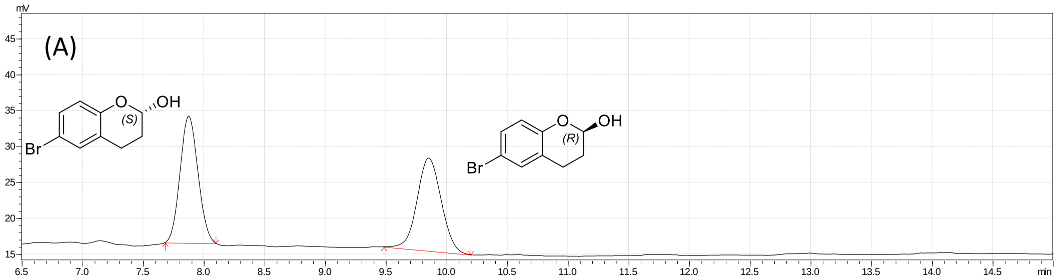

Peak#	Ret. Time [min]	Area [mV*min]	Hight [mV]	Area (%)
1	7.995	181160	17665	50.305
2	9.855	178966	12940	49.695

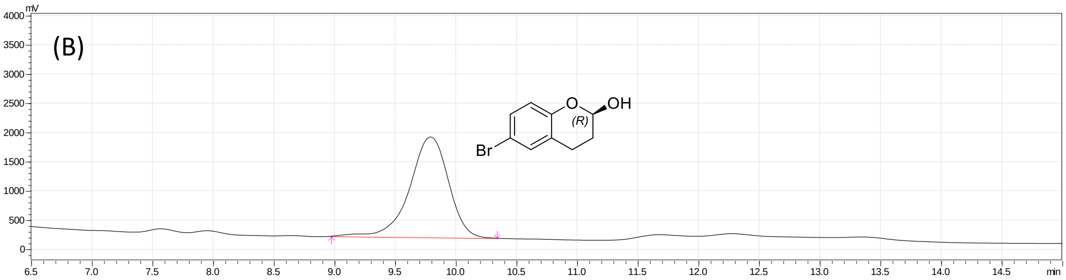

Peak#	Ret. Time [min]	Area [mV*min]	Hight [mV]	Area (%)
1	9.796	38262242	1719113	100.000

Supplementary Fig. 35. Representative chiral HPLC chromatogram of (A) rac-**11a** and (B) reaction mixture after 24 h.

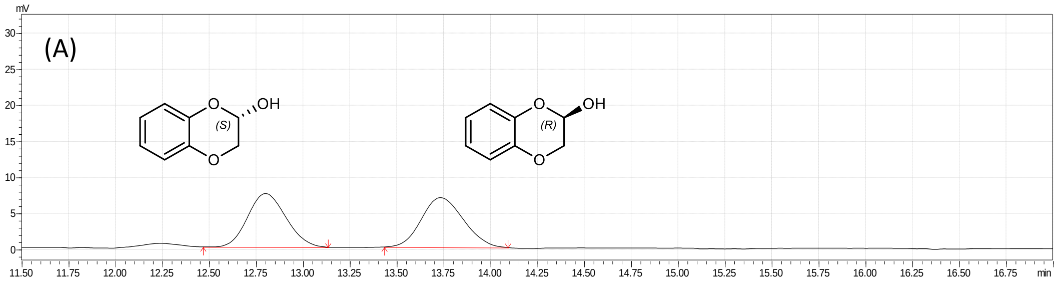

Peak#	Ret. Time [min]	Area [mV*min]	Hight [mV]	Area (%)
1	12.801	105233	7427	49.595
2	13.734	106951	6871	50.405

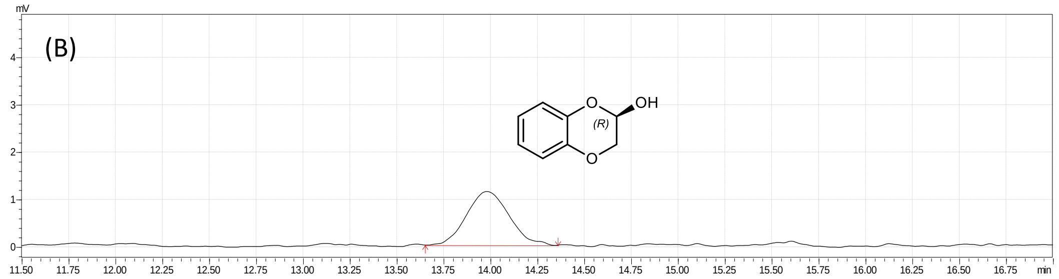

Peak#	Ret. Time [min]	Area [mV*min]	Hight [mV]	Area (%)
1	13.981	17782	1131	100.000

Supplementary Fig. 36. Representative chiral HPLC chromatogram of (A) rac-**12a** and (B) the reaction mixture after esterification (Acetic anhydride).

2.4 Supplementary data for quantum chemical calculations

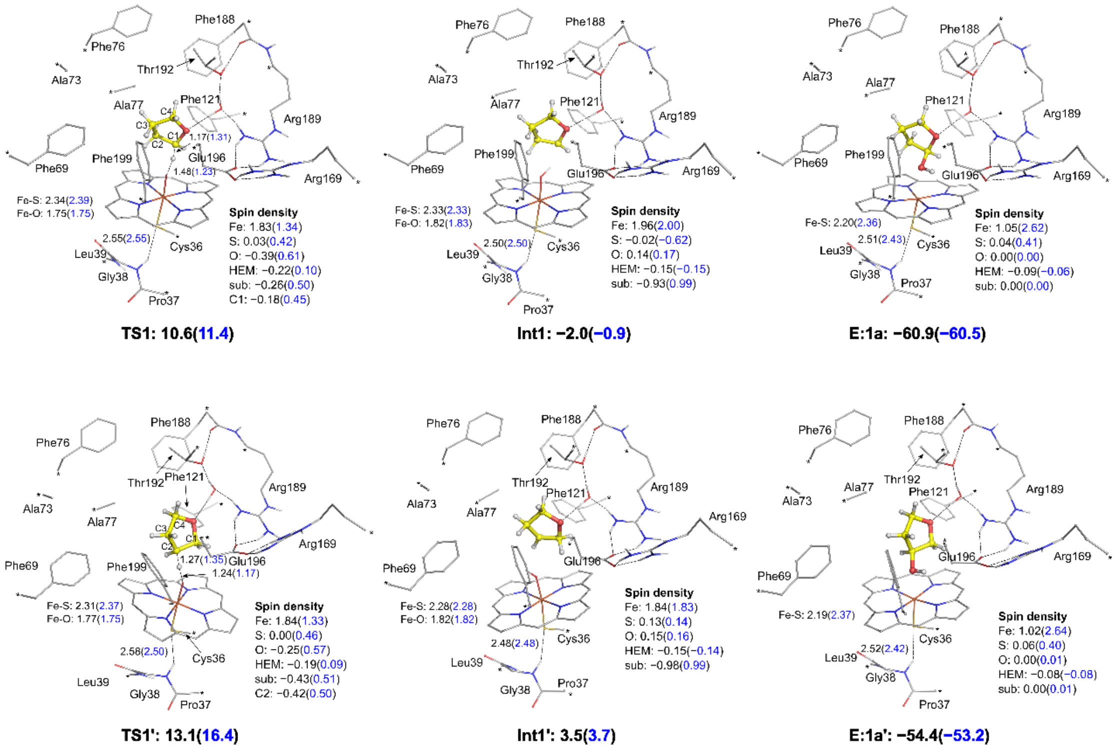

Supplementary Fig. 37. Optimized structures of the transition states and intermediates in the reaction pathway from **1** to **1a**. The "*" represents the atoms fixed in the geometry optimization. The energies relative to **E$_{\mathbf{Cpd\ I}}$:1** are also given (in kcal/mol). Numbers in black represent the relative energies at the doublet state, and numbers in blue represent the relative energies at the quartet state. Non-polar hydrogens of the protein and heme are hidden for clarity.

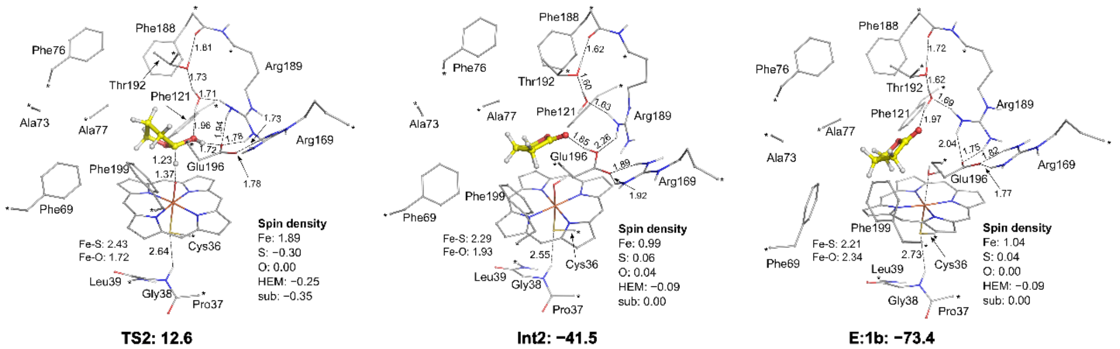

Supplementary Fig. 38. Optimized structures of the transition states and intermediates in the reaction pathway from **1a** to **1b**. The "*" represents the atoms fixed in the geometry optimization. The energies relative to **E$_{\mathbf{Cpd\ I}}$:1a** are also given (in kcal/mol). Non-polar hydrogens of the protein and heme are hidden for clarity.

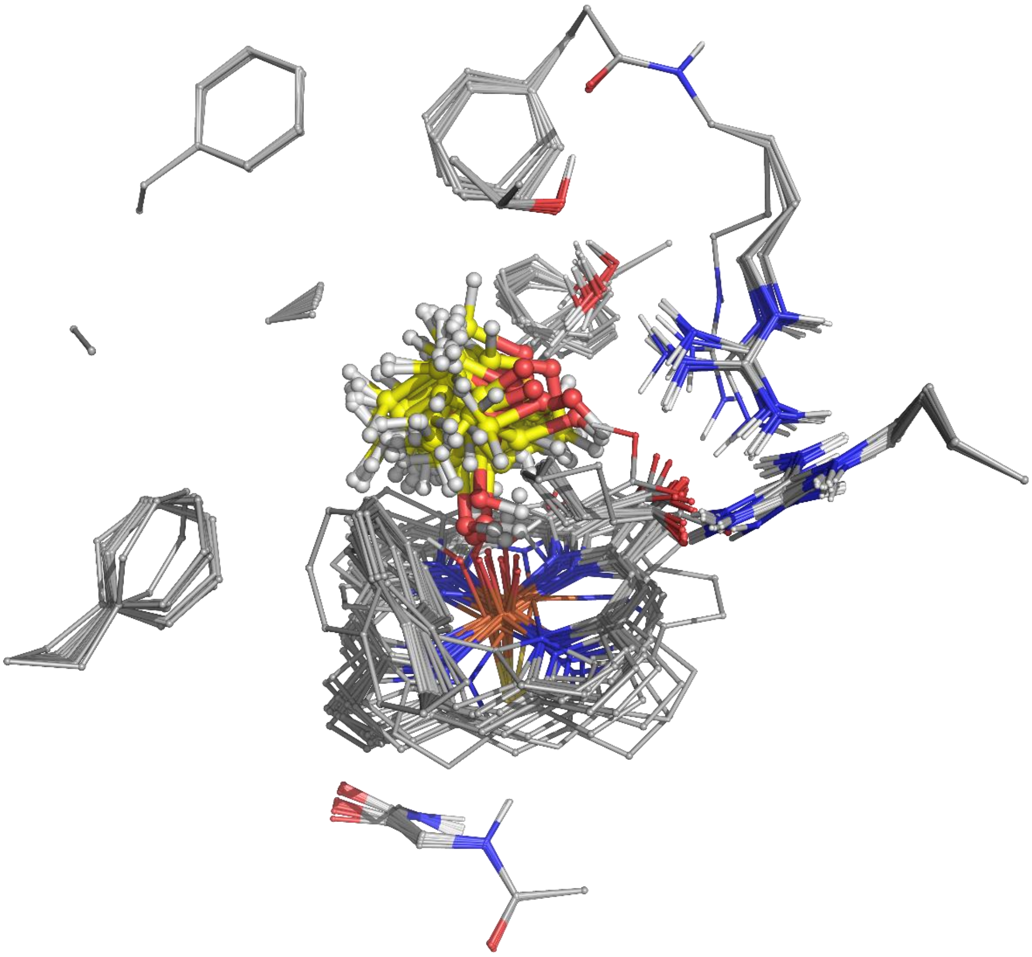

Supplementary Fig. 39. Superposition of all the optimized structures of the intermediates and transition states involved in the reaction pathway of r*Aae*UPO-catalyzed reaction.

2.5 Supplementary data for molecular dynamics simulations

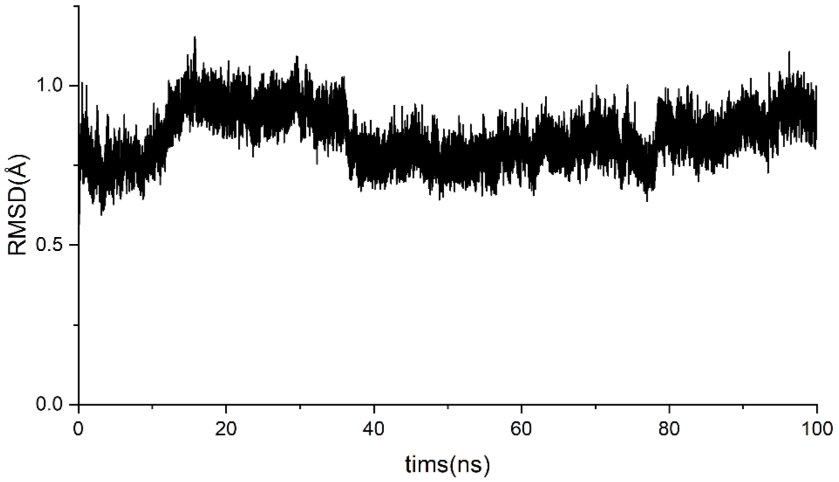

Supplementary Fig. 40. Time evolution of the root mean square deviations (RMSD) for the protein backbone during the MD simulations for the system in tetrahydrofuran solution.

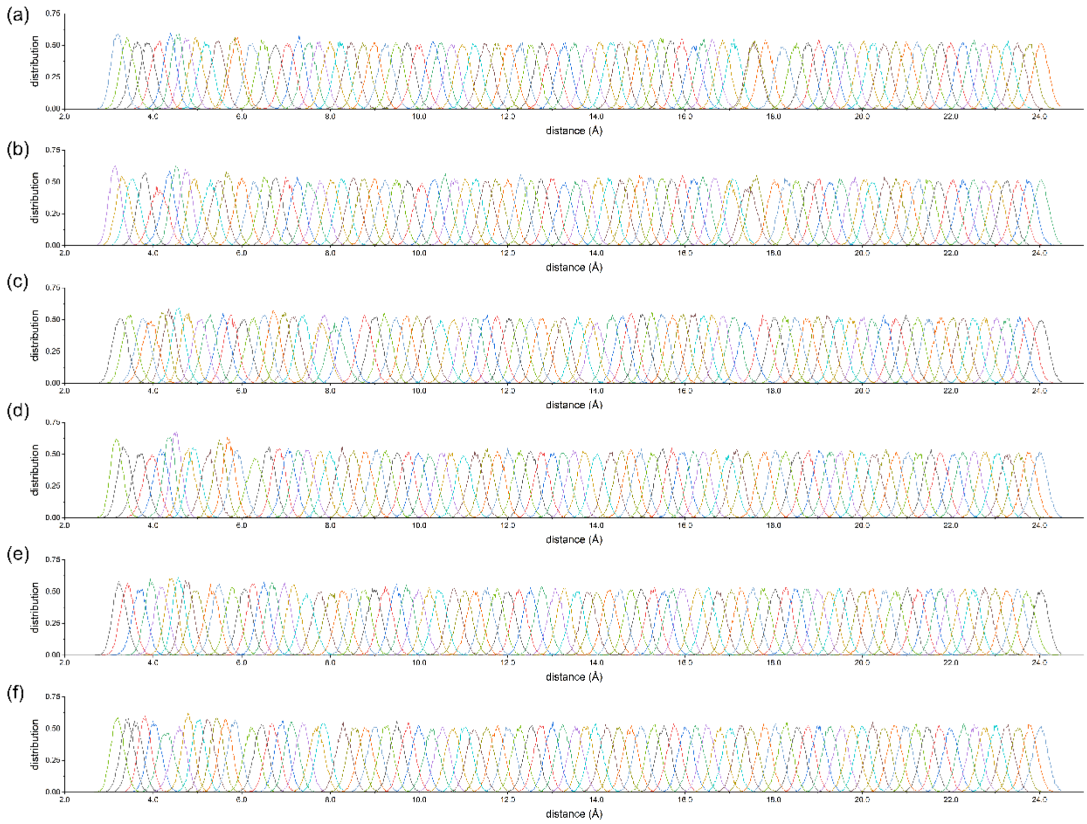

Supplementary Fig. 41. Distribution histograms of Umberlla sampling window: (a) pH=4, **1**; (b) pH=4, **1a**; (c) pH=9, **1**; (d) pH=9, **1a**; (e) pH=9, **1**, buffer; (f) pH=9, **1a**, buffer. Different colors are used to distinguish different sampling windows.

2.6 NMR spectra of chemically synthesised standards

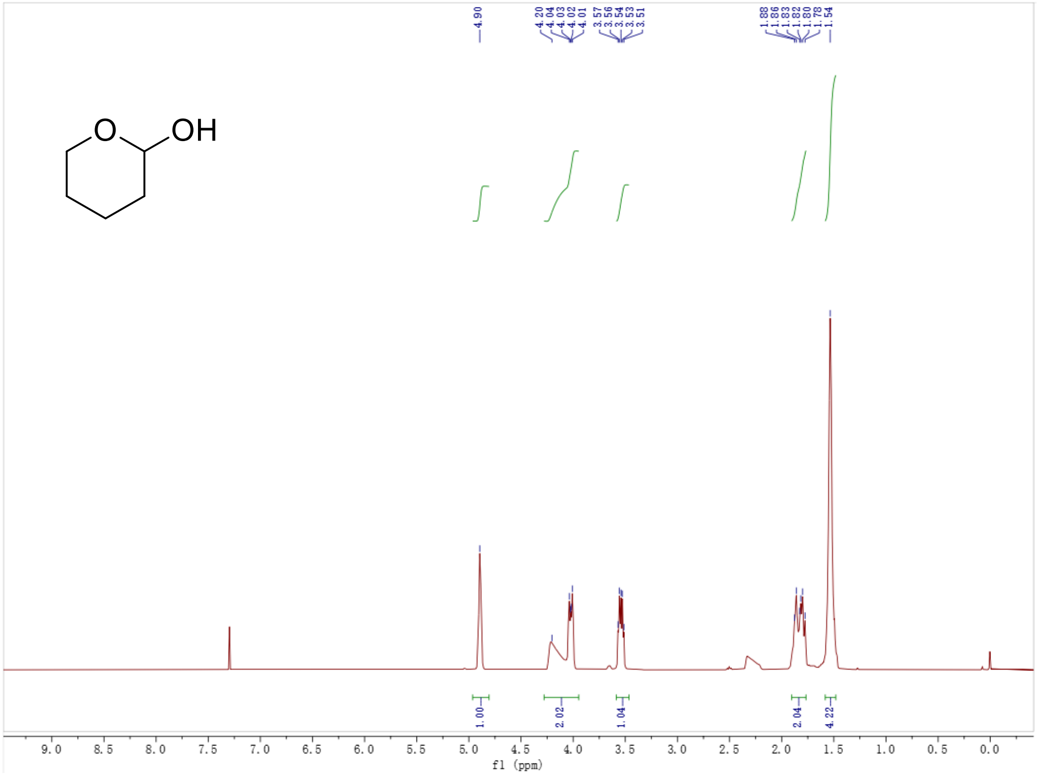

Supplementary Fig. 42. ^{1}H NMR spectrum of **2a** in $CDCl_3$.

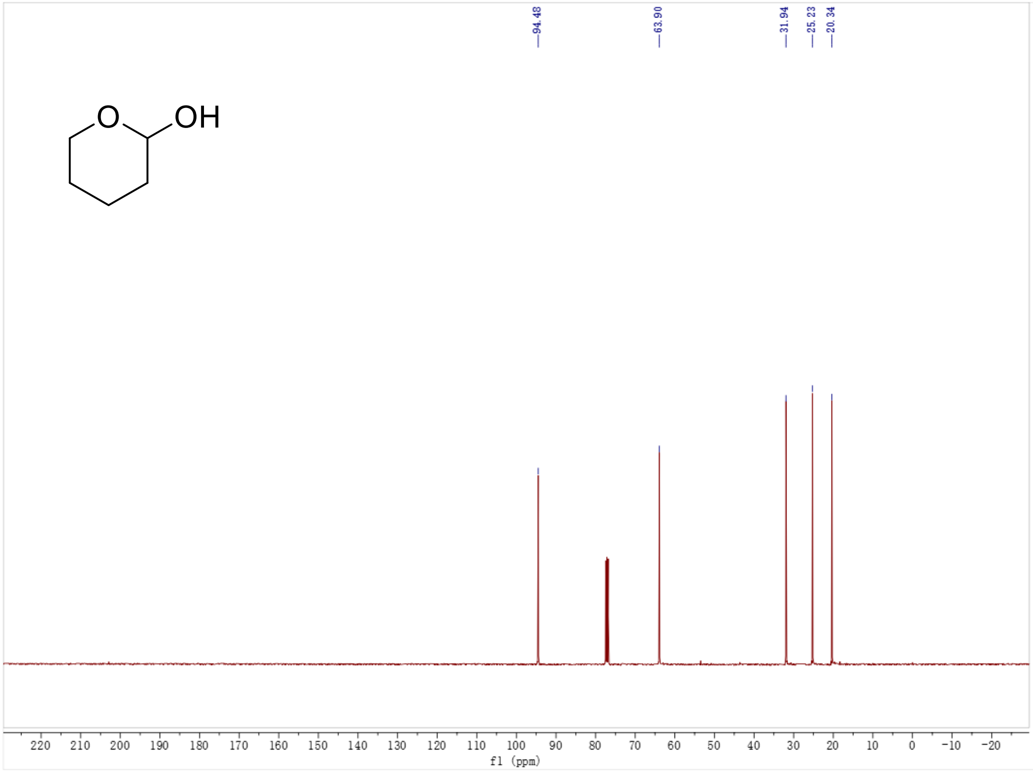

Supplementary Fig. 43. ^{13}C NMR spectrum of **2a** in $CDCl_3$.

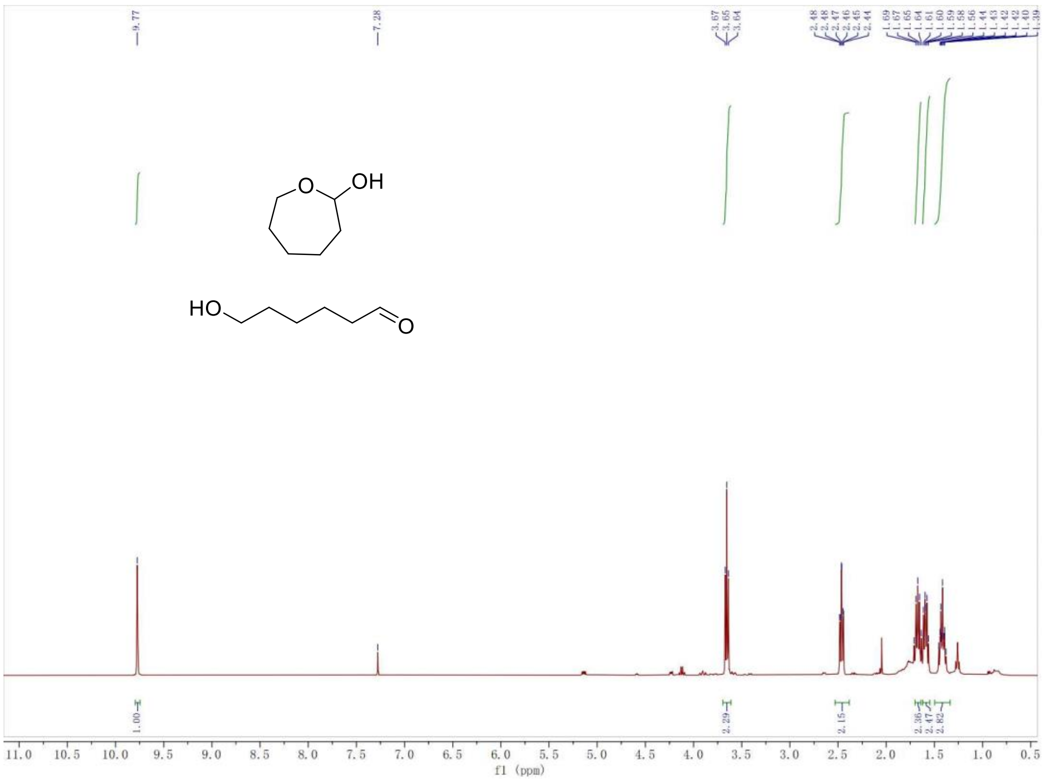

Supplementary Fig. 44. ^{1}H NMR spectrum of **3a** in $CDCl_3$.

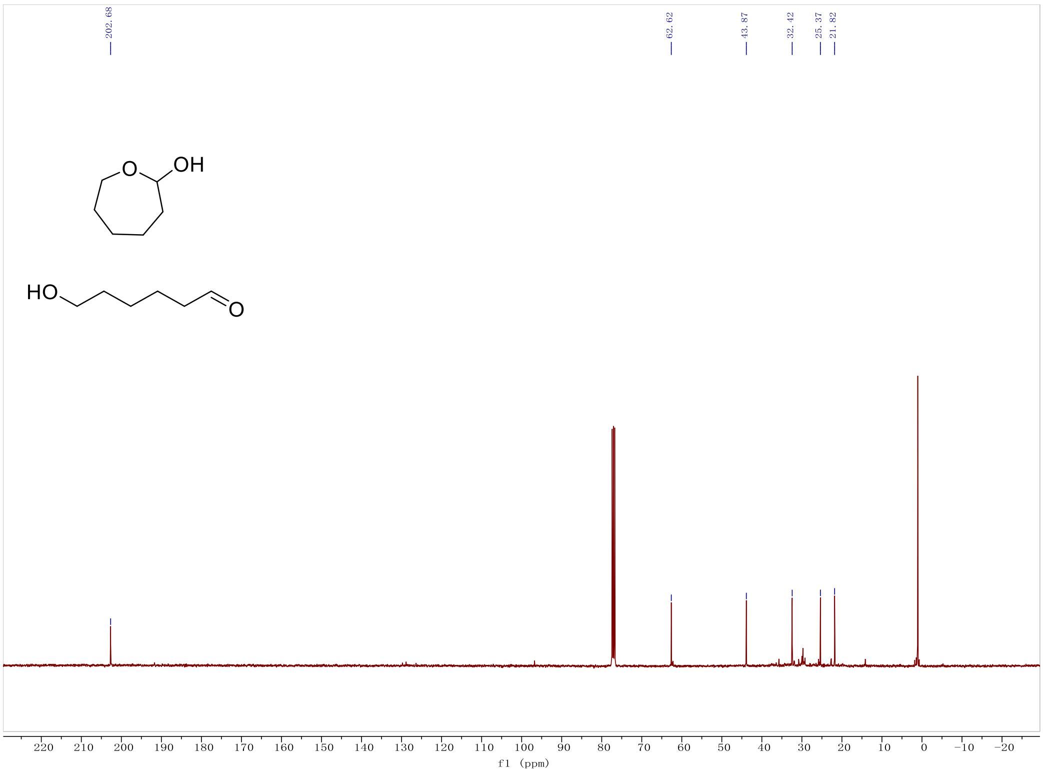

Supplementary Fig. 45. ^{13}C NMR spectrum of **3a** in $CDCl_3$.

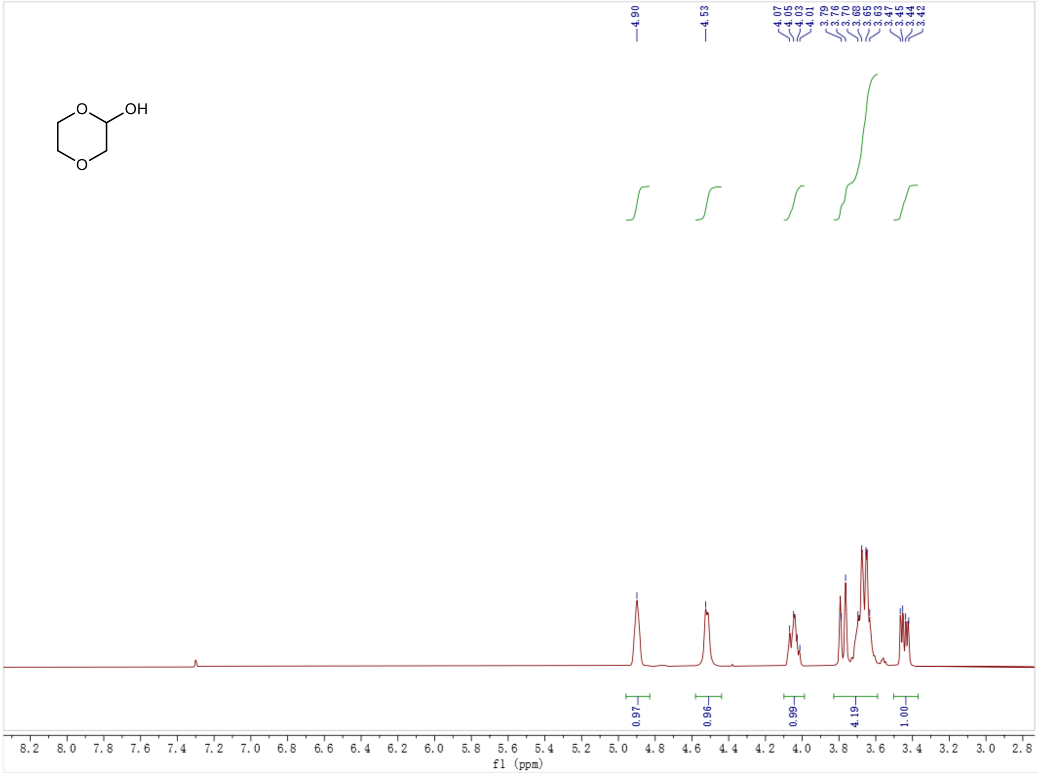

Supplementary Fig. 46. ^{1}H NMR spectrum of **5a** in $CDCl_3$.

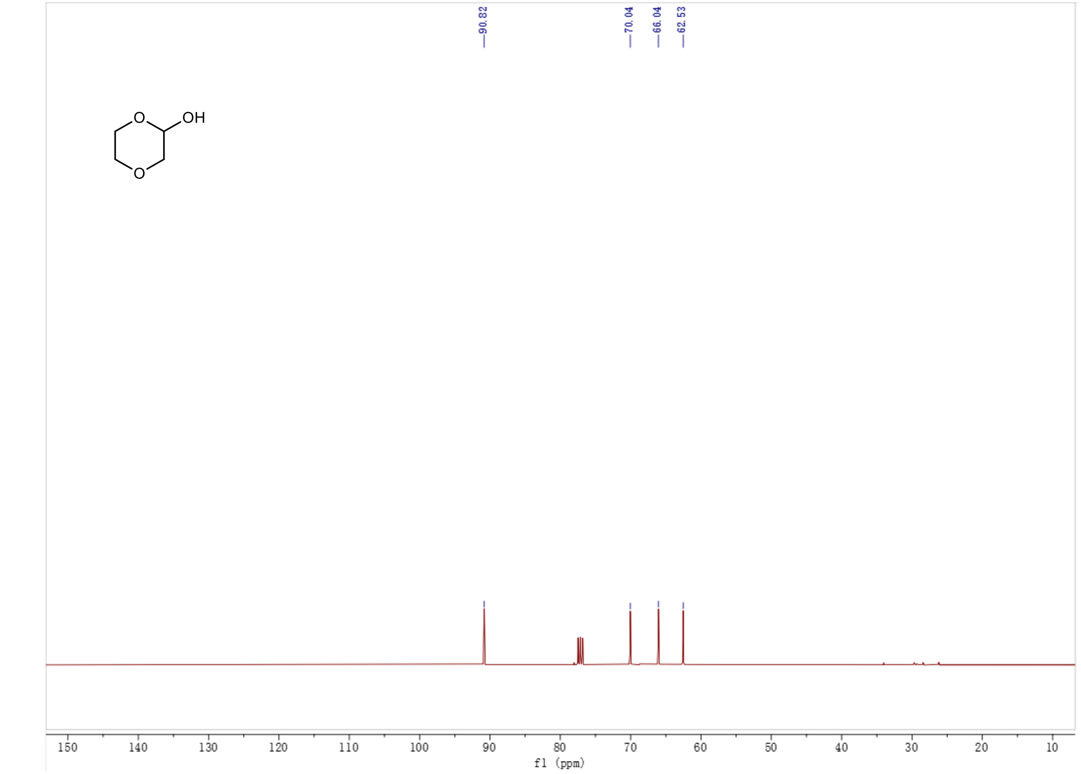

Supplementary Fig. 47. ^{13}C NMR spectrum of **5a** in $CDCl_3$.

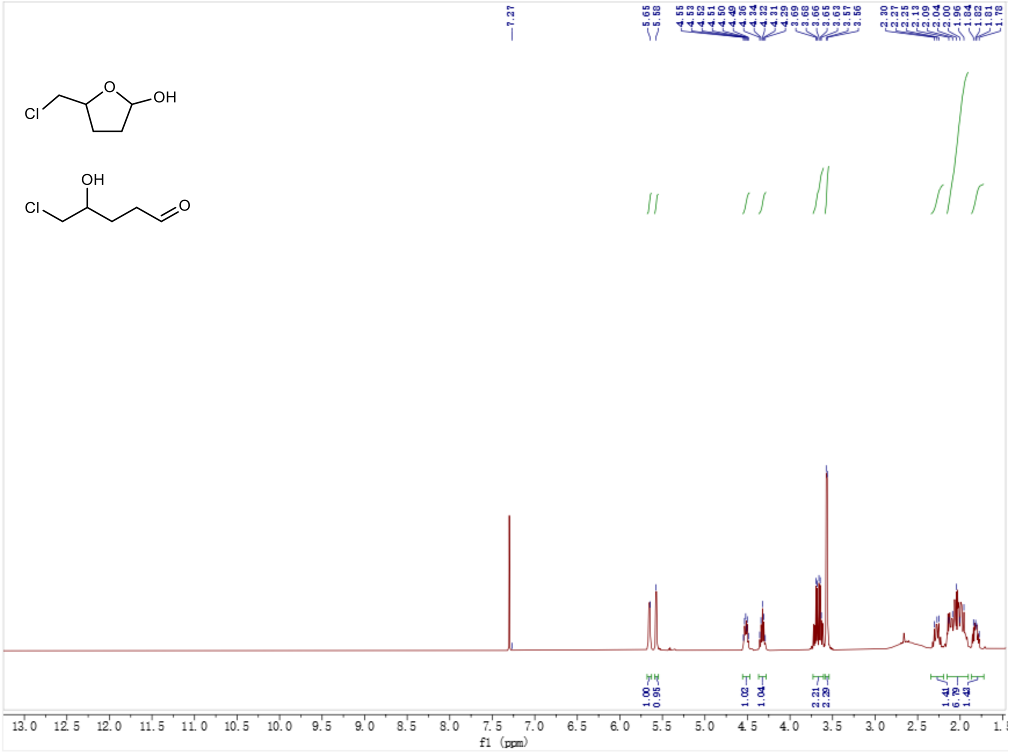

Supplementary Fig. 48. ^{1}H NMR spectrum of **6a** in $CDCl_3$.

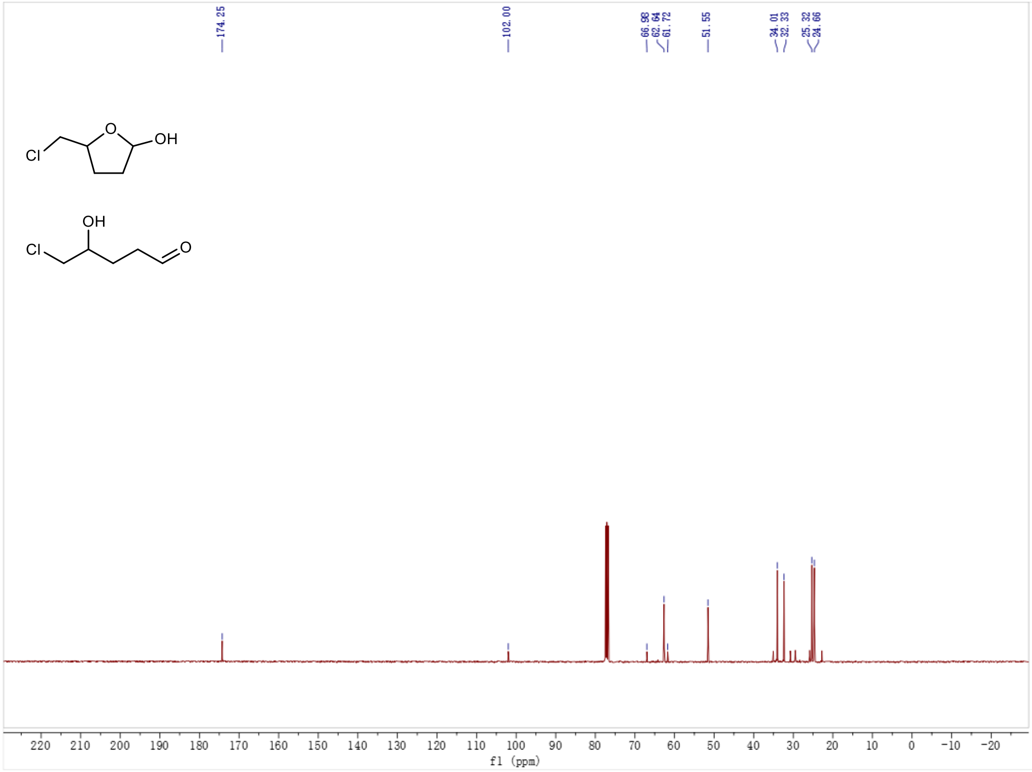

Supplementary Fig. 49. ^{13}C NMR spectrum of **6a** in $CDCl_3$.

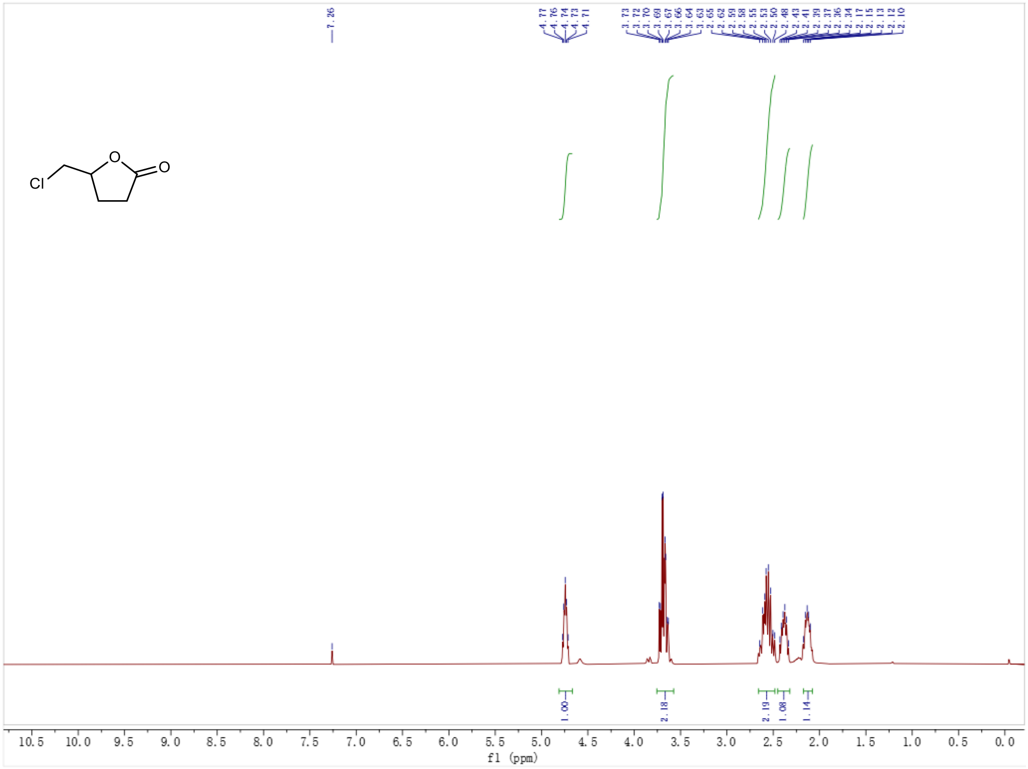

Supplementary Fig. 50. ^{1}H NMR spectrum of **6b** in $CDCl_3$.

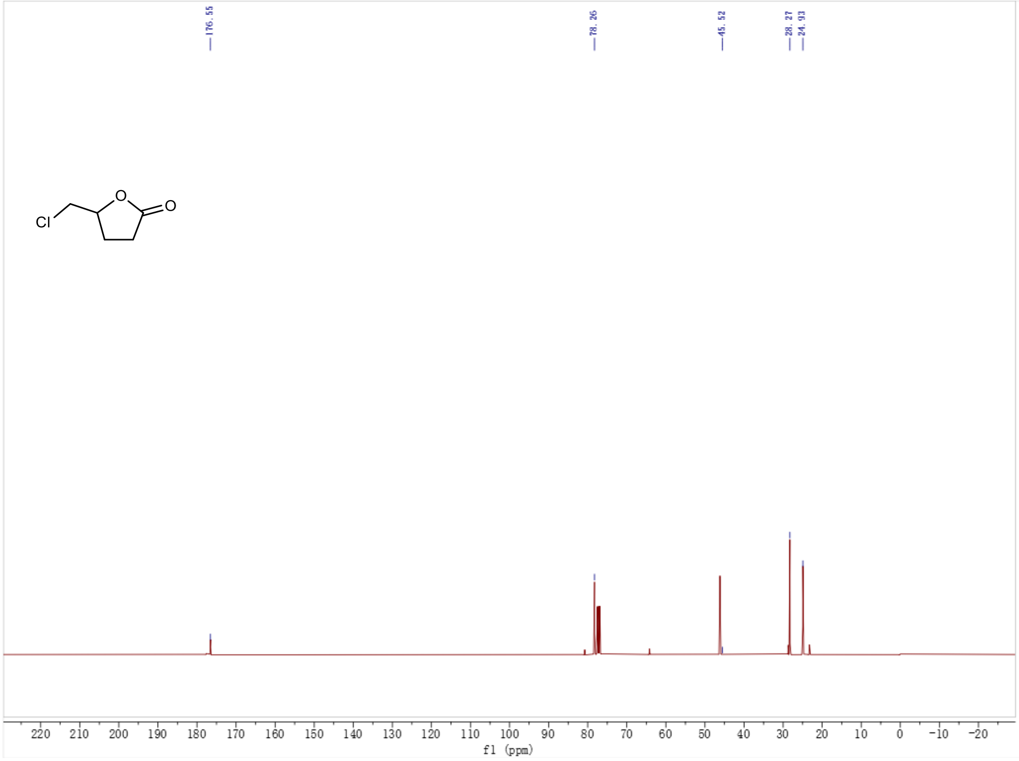

Supplementary Fig. 51. ^{13}C NMR spectrum of **6b** in $CDCl_3$.

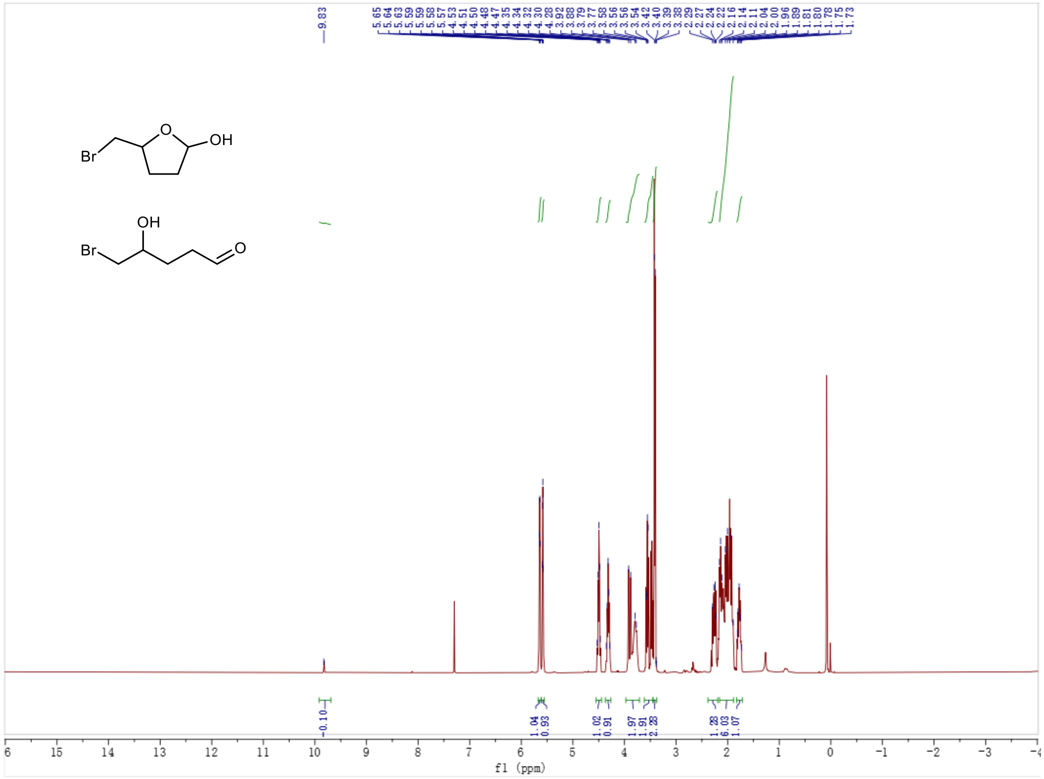

Supplementary Fig. 52. ^{1}H NMR spectrum of **7a** in $CDCl_3$.

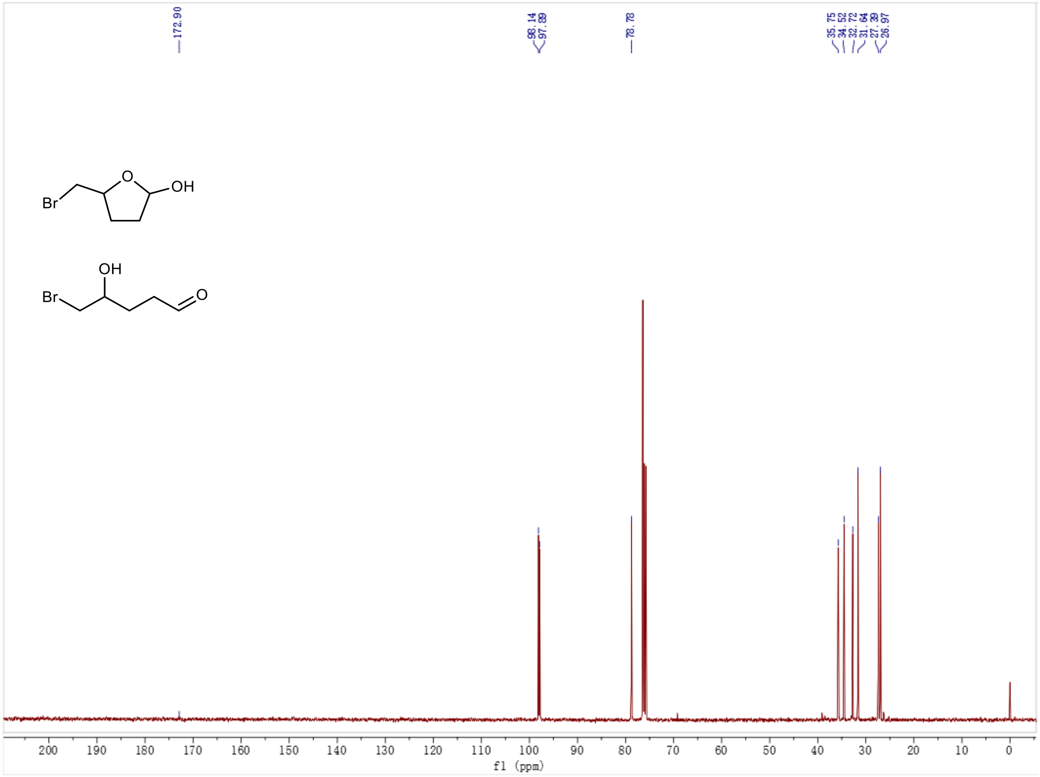

Supplementary Fig. 53. ^{13}C NMR spectrum of **7a** in $CDCl_3$.

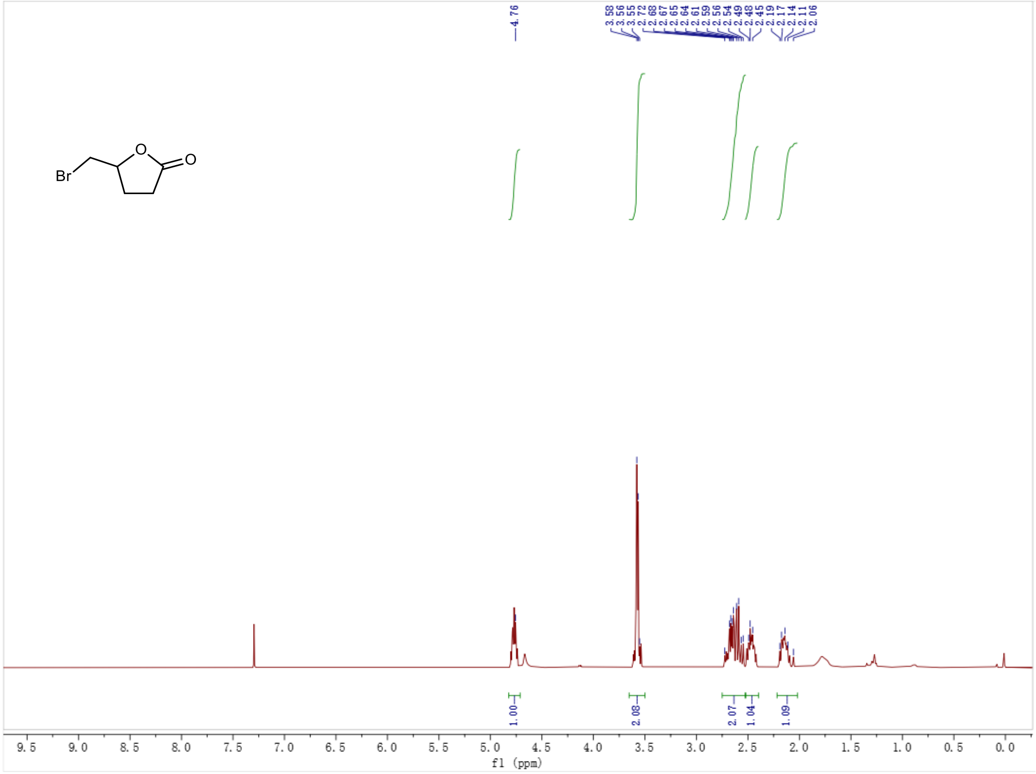

Supplementary Fig. 54. ^{1}H NMR spectrum of **7b** in $CDCl_3$.

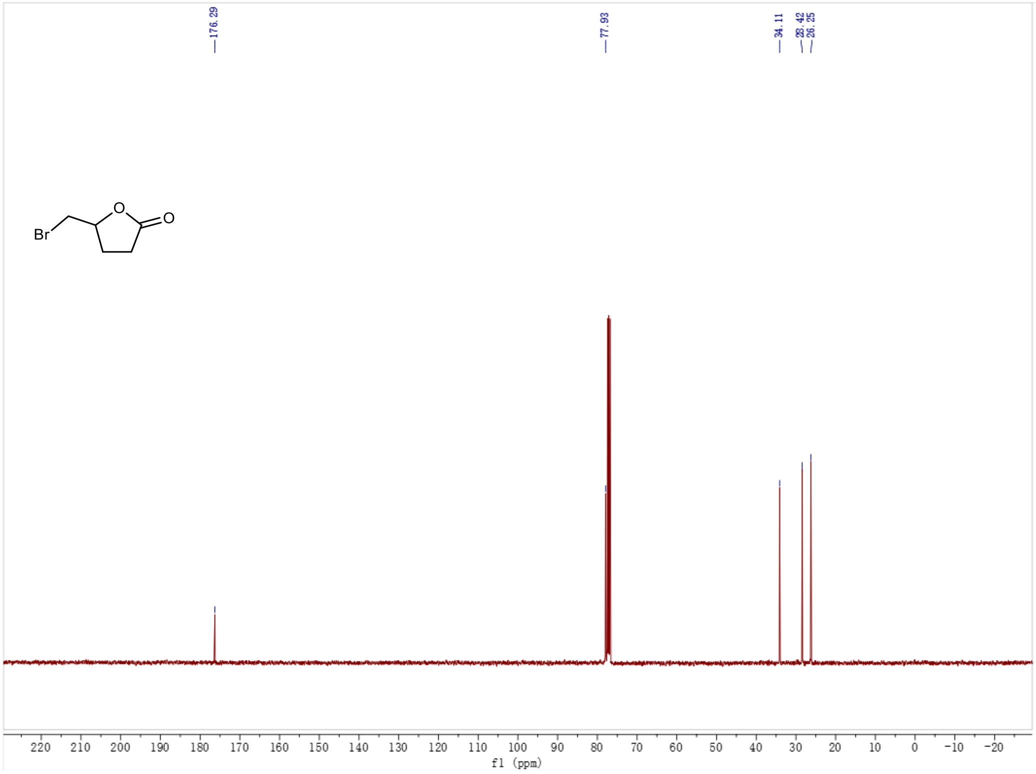

Supplementary Fig. 55. ^{13}C NMR spectrum of **7b** in $CDCl_3$.

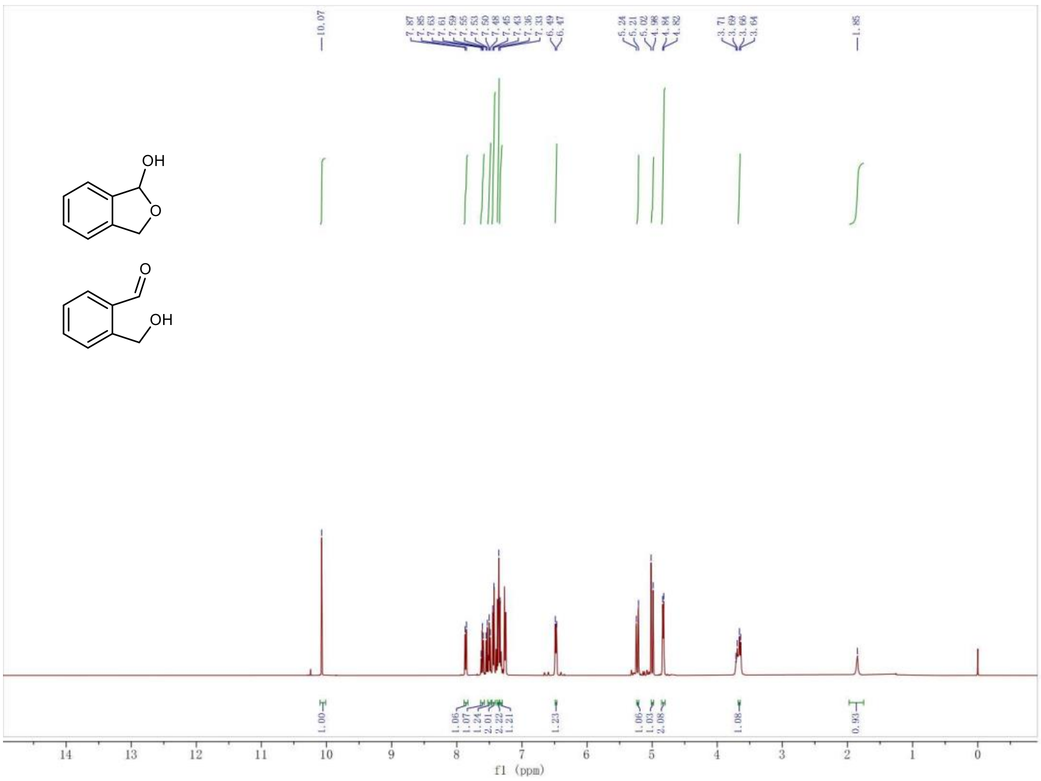

Supplementary Fig. 56. ^{1}H NMR spectrum of **8a** in $CDCl_3$.

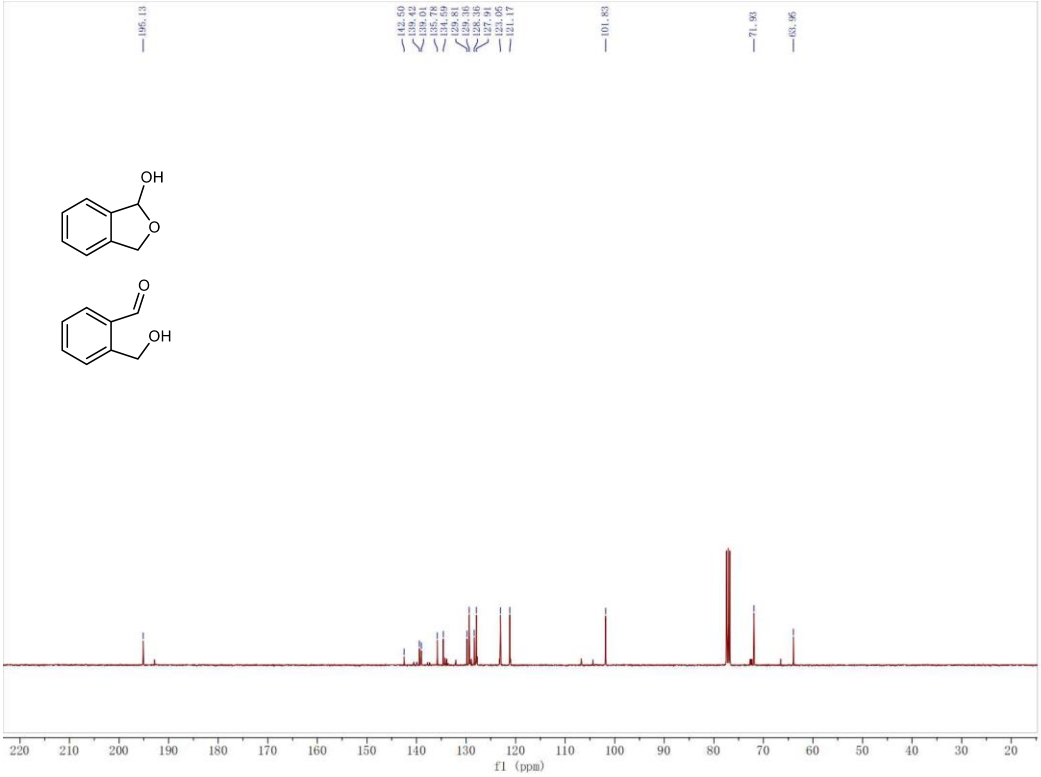

Supplementary Fig. 57. ^{13}C NMR spectrum of **8a** in $CDCl_3$.

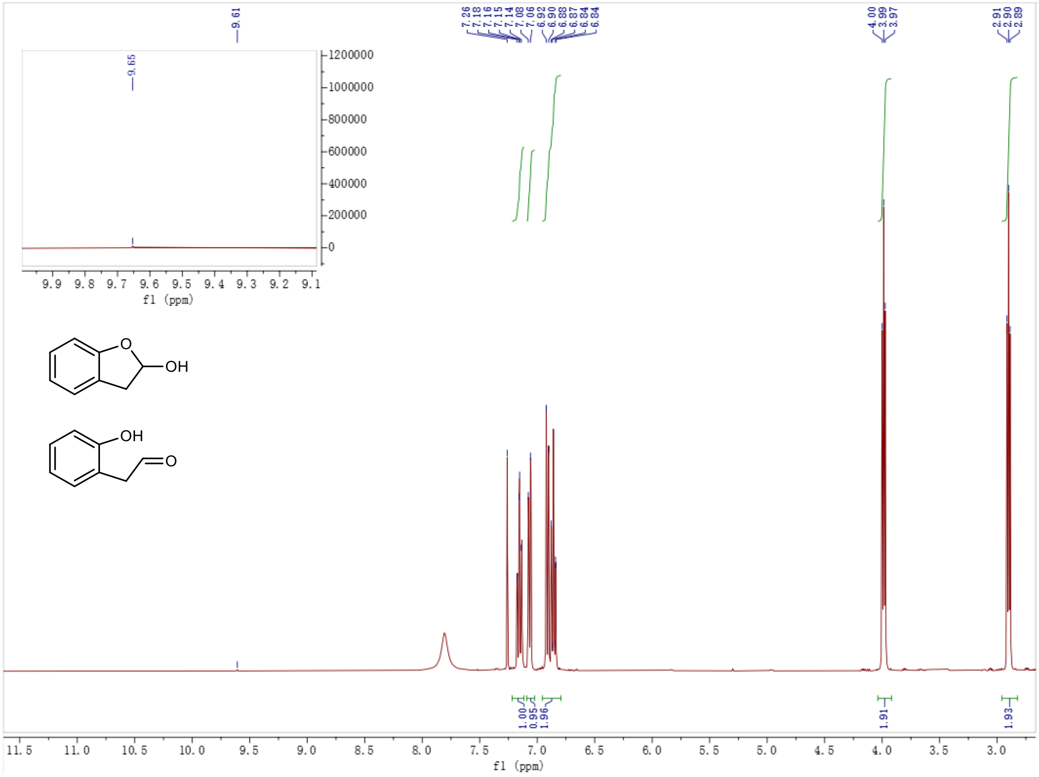

Supplementary Fig. 58. ^{1}H NMR spectrum of **9a** in $CDCl_3$.

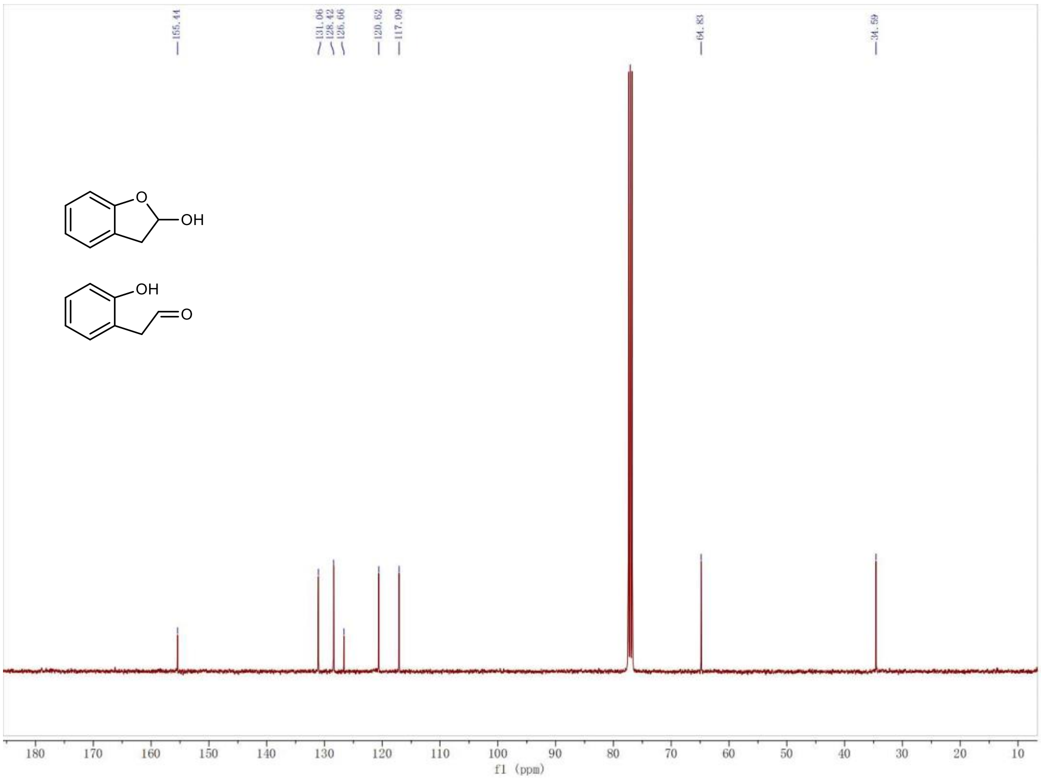

Supplementary Fig. 59. ^{13}C NMR spectrum of **9a** in $CDCl_3$.

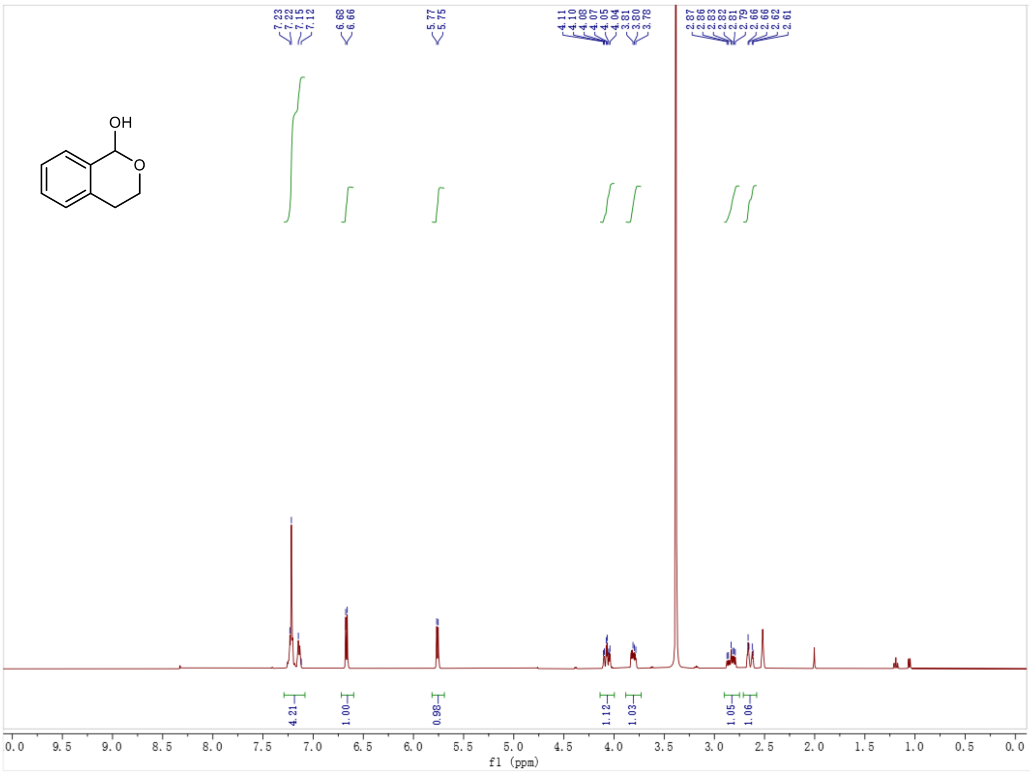

Supplementary Fig. 60. ^{1}H NMR spectrum of **10a** in DMSO-d_6.

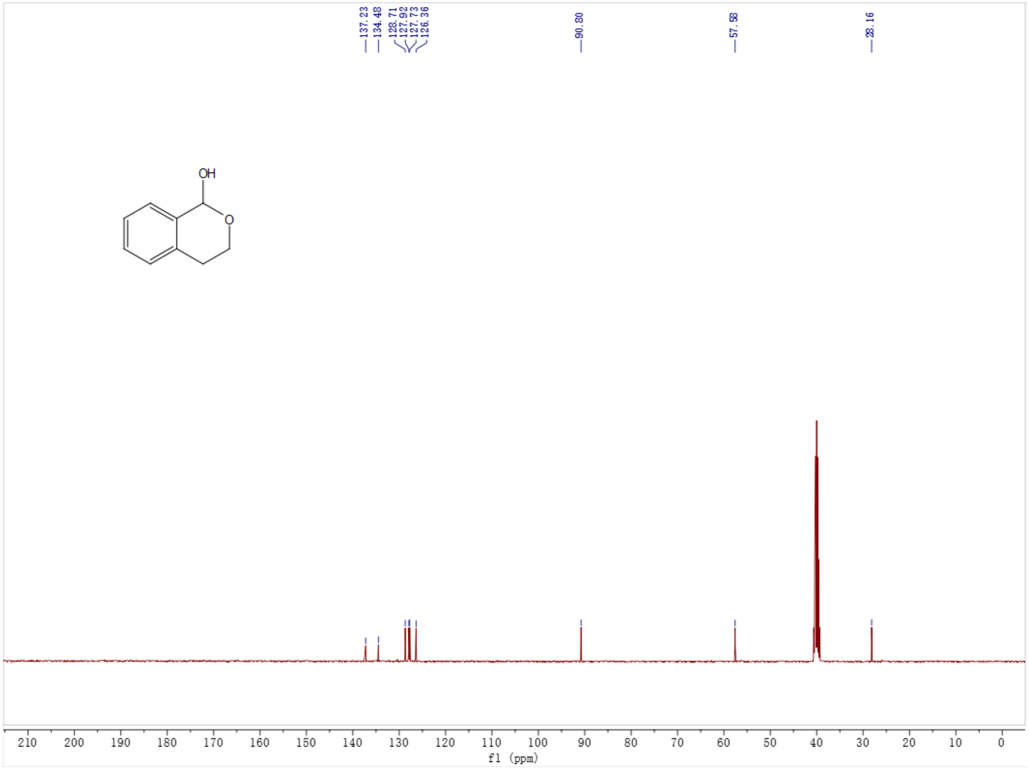

Supplementary Fig. 61. ^{13}C NMR spectrum of **10a** in DMSO-d_6.

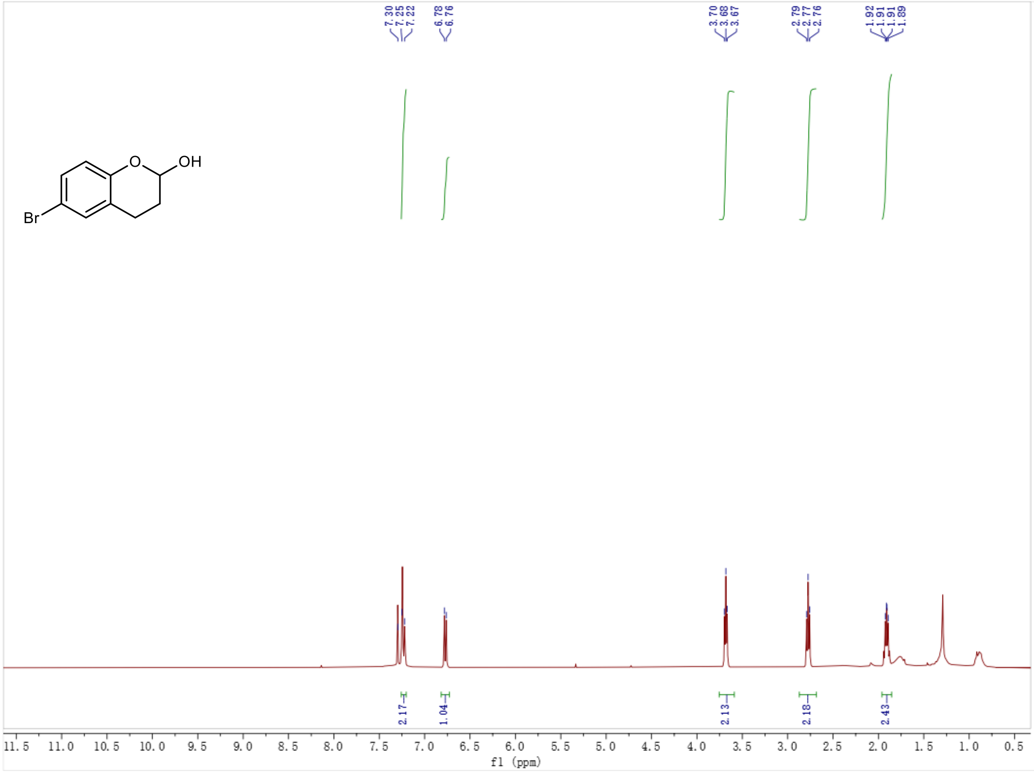

Supplementary Fig. 62. ^{1}H NMR spectrum of **11a** in $CDCl_3$.

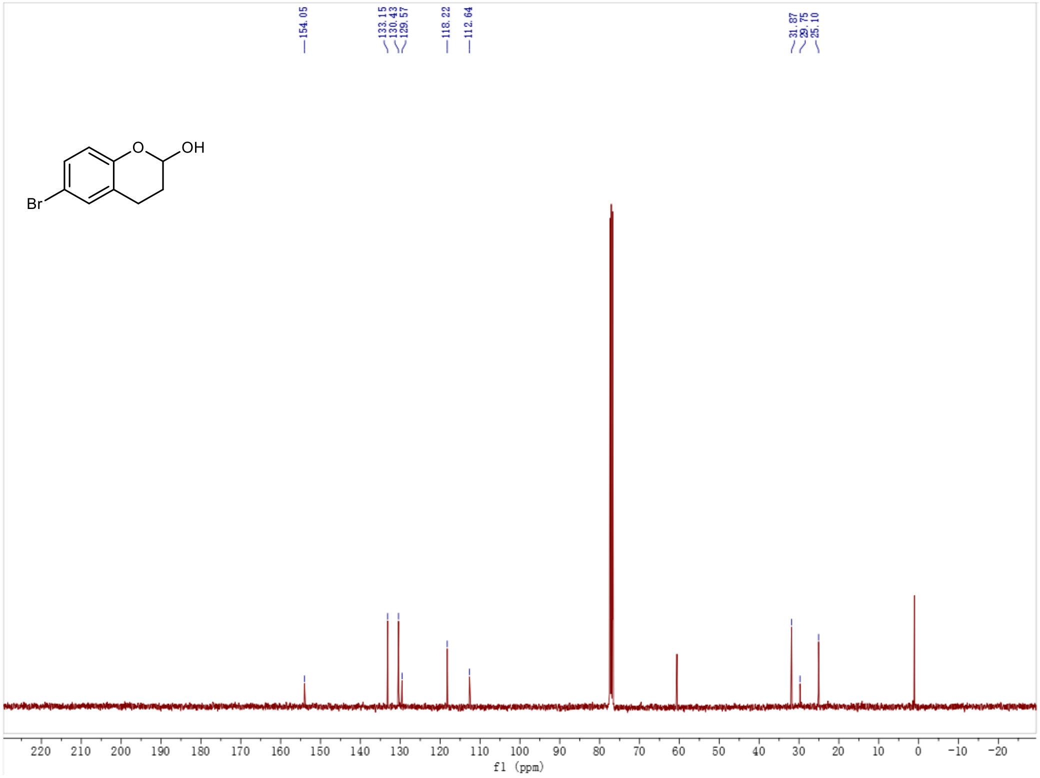

Supplementary Fig. 63. ^{13}C NMR spectrum of **11a** in $CDCl_3$.

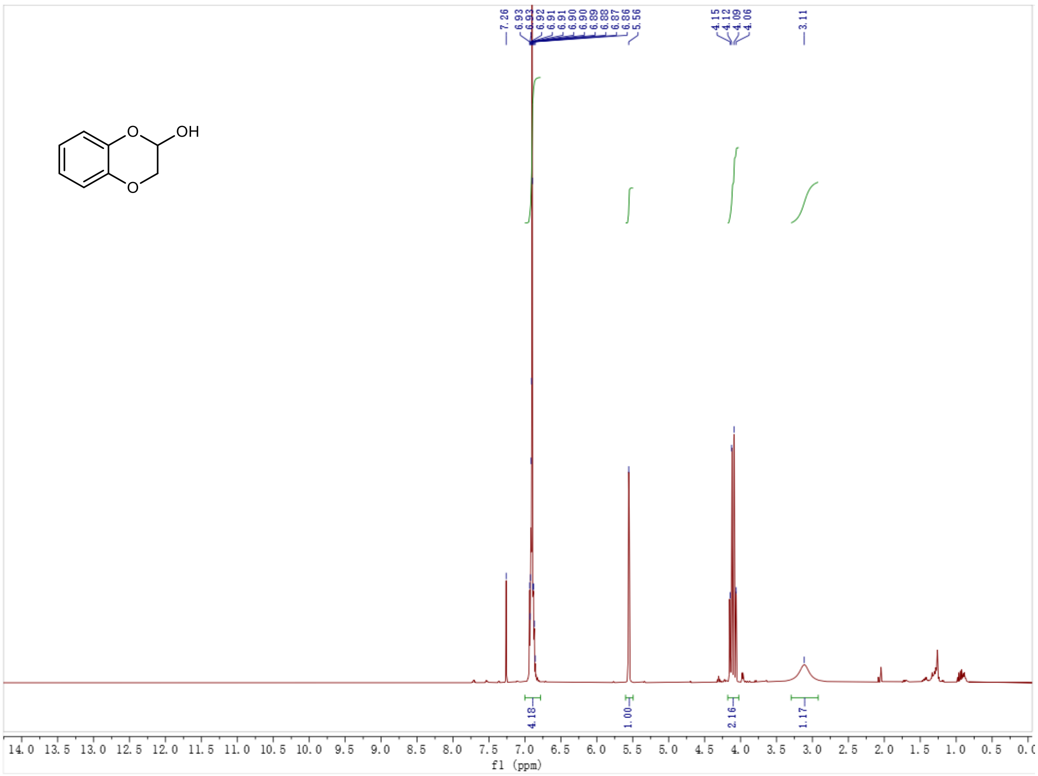

Supplementary Fig. 64. ^{1}H NMR spectrum of **12a** in $CDCl_3$.

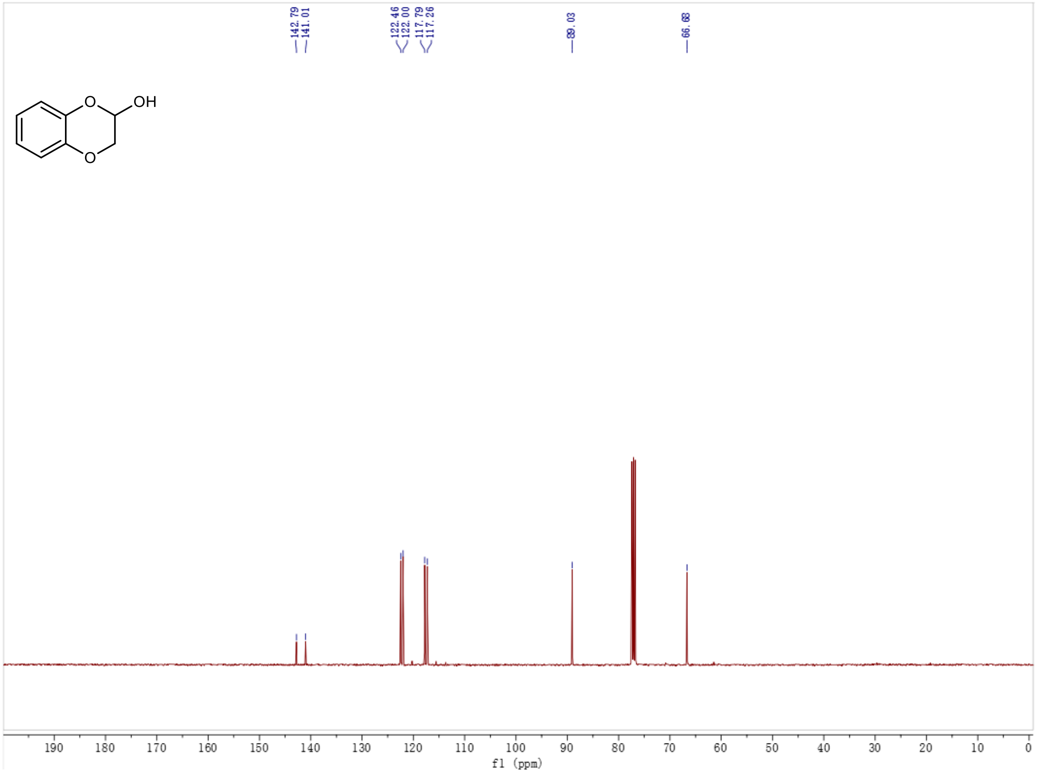

Supplementary Fig. 65. ^{13}C NMR spectrum of **12a** in $CDCl_3$.

3. Supplementary Tables

3.1 Supplementary Table 1. Enzyme concentration determined by immobilization

Resin type	[r*Aae*UPO]$_{\text{before loading}}$ (μM)	[r*Aae*UPO]$_{\text{after loading}}$ (μM)	Residual concentration (μM)	Loading ratio (%)
**LX 600**	16.67	11.74	4.93	30
**LX 603**	16.67	10.39	6.28	38
**LX 609**	16.67	11.54	5.13	31
**LX 700**	16.67	10.91	5.76	35
**LX 703**	16.67	12.30	4.37	26
**LX 704**	16.67	11.82	4.85	29
**LX 1000**	16.67	11.51	5.16	31

3.2 Supplementary Table 2. Noncycloethers catalyzed by immobilized r*Aae*UPO

Substrates	Products	
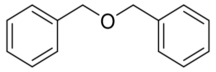 (Benzyl ether)	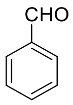 (43 mM)	
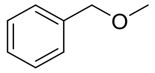 (Benzyl methyl ether)	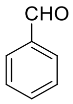 (10.3 mM)	MeOH (3.1 mM)

Conditions: [substrates] = 0.5 mL, [immobilized r*Aae*UPO] = 100 mg or 1.45 µM, [H_2O_2] = 6 mM h^{-1}, 30 °C, 24 h.

3.3 Supplementary Table 3. NMR data of the obtained hemiacetal and lactone products

Compound	NMR data
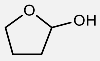<br>**tetrahydrofuran-2-ol (1a)**	^{1}H NMR (400 MHz, DMSO-d_6) δ (ppm) 5.87 (d, *J* = 4 Hz, 1H), 5.34-5.32 (m, 1H), 3.84-3.80 (m, 1H), 3.70-3.67 (m, 1H), 1.90-1.65 (m, 4H).
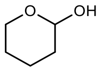<br>**tetrahydropyran-2-ol (2a)**	^{1}H NMR (400 MHz, $CDCl_3$) δ (ppm) 4.90 (s, 1H), 4.20-4.01 (m, 2H), 3.57-3.51 (m, 1H), 1.88-1.78 (m, 1H), 1.54 (s, 1H).<br><br>^{13}C NMR (101 MHz, $CDCl_3$) δ (ppm) 94.48 (s), 63.90 (s), 31.94 (s), 25.23 (s), 20.34 (s).
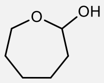<br>**oxepan-2-ol (3a)**	^{1}H NMR (400 MHz, $CDCl_3$) δ (ppm) 9.77 (s, 1H), 3.65 (t, *J* = 8 Hz, 2H), 2.47 (m, 2H), 1.65 (m, 2H), 1.58 (m, 2H), 1.40 (m, 2H).<br><br>^{13}C NMR (101 MHz, $CDCl_3$) δ (ppm) 202.68 (s), 62.62 (s), 43.87 (s), 32.42 (s), 25.37 (s), 21.82 (s).
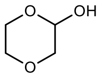<br>**1,4-Dioxan-2-ol (5a)**	^{1}H NMR (400 MHz, $CDCl_3$) δ (ppm) 4.90 (s, 1H), 4.53 (s, 1H), 4.07-4.01 (m, 1H), 3.79-3.63 (m, 4H), 3.47- 3.42 (m, 1H).<br><br>^{13}C NMR (101 MHz, $CDCl_3$) δ (ppm) 90.82 (s), 70.04 (s), 66.04 (s), 62.53 (s).
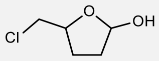<br>**5-Chloromethyl-tetrahydro-furan-2-ol (6a)**	^{1}H NMR (400 MHz, $CDCl_3$) δ (ppm) 5.65 (s, 1H), 5.58 (s, 1H), 4.49-4.55(m, 1H), 4.29-4.36(m, 1H), 3.63-3.69(m, 2H), 3.56 (d, *J* = 4 Hz, 2H), 2.25-2.30(m, 1H), 1.96-2.13(m, 6H), 1.78-1.84(m, 1H).<br><br>^{13}C NMR (101 MHz, $CDCl_3$) δ (ppm) 97.80 (s), 80.66 (s), 48.29 (s), 47.00 (s), 32.81 (s), 33.86 (s), 27.37 (s), 25.99 (s)

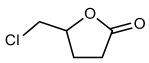<br>**5-Chloromethyl-tetrahydro-furan-2-one (6b)**	^{1}H NMR (400 MHz, $CDCl_3$) δ (ppm) 4.77-4.71 (m, 1H), 3.73-3.63 (m, 2H), 2.65-2.48 (m, 2H), 2.43-2.34 (m, 1H), 2.17-2.10 (m, 1H).<br><br>^{13}C NMR (101 MHz, $CDCl_3$) δ (ppm) 176.55 (s), 78.26 (s), 45.52 (s), 28.27 (s), 24.93 (s).
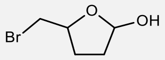<br>**5-bromomethyl-tetrahydro-furan-2-ol (7a)**	^{1}H NMR (400 MHz, $CDCl_3$) δ (ppm) 5.65-5.63 (m, 1H), 5.59-5.57 (m, 1H), 4.53-4.37 (m, 1H), 4.35-4.28 (m, 1H), 3.92-3.77 (m, 2H), 3.58-3.54 (m, 2H), 3.42-3.38 (m, 2H), 2.29-2.16 (m, 1H), 2.14-1.89 (m, 6H), 1.81-1.73 (m, 1H).<br><br>^{13}C NMR (101 MHz, $CDCl_3$) δ (ppm) 98.14 (s), 97.89 (s), 78.78 (s), 76.40 (s), 35.75 (s), 34.52 (s), 32.72 (s), 31.64 (s), 27.39 (s), 26.97 (s).
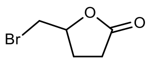<br>**5-bromomethyl-tetrahydro-furan-2-one (7b)**	^{1}H NMR (400 MHz, $CDCl_3$) δ (ppm) 4.76 (m, 1H), 3.58-3.55 (m, 2H), 2.72-2.54 (m, 2H), 2.19-2.17 (m, 1H), 2.14-2.06 (m, 1H).<br><br>^{13}C NMR (101 MHz, $CDCl_3$) δ (ppm) 176.29 (s), 77.93 (s), 34.11 (s), 28.42 (s), 26.25 (s).
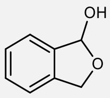<br>**1,3-dihydroisobenzofuran-1-ol (8a)**	^{1}H NMR (400 MHz, $CDCl_3$) δ (ppm) 1.85 ((s, aldehyde-1H), 3.64-3.71 (m, lactol-1H), 4.82 (d, *J* = 8 Hz, aldehyde-2H), 4.98 (d, *J* = 8 Hz, lactol-1H), 5.21 (d, 12 Hz, lactol-1H), 6.47 (dd, *J* = 8.0 Hz, lactol-1H), 7.33-7.87 (m, lactol/aldehyde-8H) 10.07 (s, aldehyde-1H).<br><br>^{13}C NMR (101 MHz, $CDCl_3$) δ (ppm) 195.13 (s), 142.50 (s), 139.42 (s), 139.01 (s), 135.78 (s), 134.59 (s), 129.81 (s), 129.36 (s), 128.36 (s), 127.91 (s), 123.05 (s), 121.17 (s), 101.83 (s), 71.93 (s), 63.95 (s).
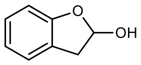<br>**2,3-dihydrobenzofuran-2-ol (9a)**	^{1}H NMR (400 MHz, $CDCl_3$) δ (ppm) 1.85 (s, 1H), 3.64-3.71 (m, 1H), 4.82 (d, *J* = 8 Hz, 2H), 4.98 (d, *J* = 8 Hz, 1H), 5.21 (d, *J* =12 Hz, 1H), 6.47 (dd, *J* = 8.0 Hz, 1H), 7.33-7.87 (m, 8H) 10.07 (s, 1H).<br><br>^{13}C NMR (101 MHz, $CDCl_3$) δ (ppm) 195.13 (s), 142.50 (s), 139.42 (s), 139.01 (s), 135.78 (s), 134.59 (s), 129.81 (s), 129.36 (s), 128.36 (s), 127.91 (s), 123.05 (s), 121.17 (s), 101.83 (s),

	71.93 (s), 63.95 (s).
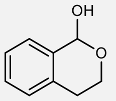<br>**isochroman-1-ol (10a)**	^{1}H NMR (400 MHz, $CDCl_3$) δ (ppm) 4.25 (m, 2H), 6.76 (d, *J* = 8 Hz, 1H), 3.70-3.67 (m, 2H), 3.79-3.76 (m, 2H), 1.92- 1.89 (m, 2H).<br><br>^{13}C NMR (101 MHz, DMSO-d_6) δ (ppm) 137.23, 134.48, 128.71, 127.92, 127.73, 126.36, 90.80, 57.58, 28.16.
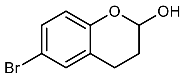<br>**6-bromo-3,4-dihydro-2H-1-benzopyran-2-ol (11a)**	^{1}H NMR (400 MHz, $CDCl_3$) δ (ppm) 4.25 (m, 2H), 6.76 (d, *J* = 8 Hz, 1H), 3.70-3.67 (m, 2H), 3.79-3.76 (m, 2H), 1.92- 1.89 (m, 2H).<br><br>^{13}C NMR (101 MHz, $CDCl_3$) δ (ppm) 154.05 (s), 133.15 (s), 130.43 (s), 129.57 (s), 118.22 (s), 112.64 (s), 31.87 (s), 29.75 (s), 25.10 (s).
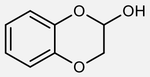<br>**2-Hydroxy-benz-1.4-dioxan (12a)**	^{1}H NMR (400 MHz, $CDCl_3$) δ (ppm) 6.93-6.86 (m, 4H), 5.56 (s, 1H), 4.15-4.06 (m, 2H), 3.11 (s, 1H).<br><br>^{13}C NMR (101 MHz, $CDCl_3$) δ (ppm) 142.79 (s), 141.01 (s), 122.46 (s), 122.00 (s), 117.79 (s), 117.26 (s), 89.03 (s), 66.68 (s).

3.4 Supplementary Table 4. Details of the gas chromatograph and temperature profiles

**Substrate**	***T*** T_R **[min]**	**Temperature profile**
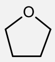	tetrahydrofuran-2-ol (**2a**)<br>4.31<br>γ-butyrolactone (**2b**)<br>5.33	80 °C holding for 2.0 min; 20 °C min^{-1} to 220 °C holding for 2.0 min; 30 °C min^{-1} to 280 °C for 0.5 min.
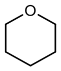	tetrahydropyran-2-ol (**2a**)<br>5.39<br>3,4,5,6-tetrahydro-2H-pyran-2-one (**2b**)<br>6.92	80 °C holding for 2.0 min; 20 °C min^{-1} to 220 °C holding for 2.0 min; 30 °C min^{-1} to 280 °C for 0.5 min.
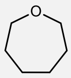	oxepan-2-ol (**3a**)<br>7.11<br>hexahydro-2H-oxepin-2-one (**3b**)<br>7.85	80 °C holding for 2.0 min; 20 °C min^{-1} to 220 °C holding for 2.0 min; 30 °C min^{-1} to 280 °C for 0.5 min.
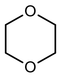	1,4-Dioxan-2-ol (**5a**)<br>5.28	80 °C holding for 2.0 min; 20 °C min^{-1} to 220 °C holding for 2.0 min; 30 °C min^{-1} to 280 °C for 0.5 min.
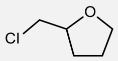	5-Chloromethyl-tetrahydro-furan-2-ol (**6a**)<br>7.22<br>5-Chloromethyl-tetrahydro-furan-2-one (**6b**)<br>8.02	80 °C holding for 2.0 min; 20 °C min^{-1} to 220 °C holding for 2.0 min; 30 °C min^{-1} to 280 °C for 0.5 min.
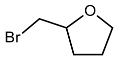	5-bromomethyl-tetrahydro-furan-2-ol (**7a**)<br>8.09<br>5-bromomethyl-tetrahydro-furan-2-one (**7b**)<br>8.83	80 °C holding for 2.0 min; 20 °C min^{-1} to 220 °C holding for 2.0 min; 30 °C min^{-1} to 280 °C for 0.5 min.
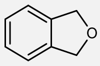	1,3-dihydroisobenzofuran-1-ol (**8a**)<br>8.95<br>1,3-dihydroisobenzofuran-1-one (**8b**)<br>10.07	80 °C holding for 2.0 min; 20 °C min^{-1} to 220 °C holding for 2.0 min; 30 °C min^{-1} to 280 °C for 0.5 min.
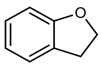	2,3-dihydrobenzofuran-2-ol (**9a**)<br>7.13<br>2,3-dihydrobenzofuran-2-one (**9b**)<br>8.81	80 °C holding for 2.0 min; 20 °C min^{-1} to 220 °C holding for 2.0 min; 30 °C min^{-1} to 280 °C for 0.5 min.
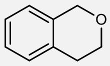	isochroman-1-ol (**10a**)<br>10.73<br>isochroman-1-one (**10b**)	80 °C holding for 2.0 min; 20 °C min^{-1} to 220 °C holding for 2.0 min;

	11.56	30 °C min^{-1} to 280 °C for 0.5 min.
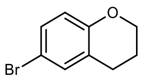	6-bromo-3,4-dihydro-2H-1-benzopyran-2-ol (**11a**) 12.76<br>6-bromo-3,4-dihydro-2H-1-benzopyran-2-one (**11b**) 13.21	80 °C holding for 1.5 min; 20 °C min^{-1} to 220 °C holding for 2.0 min; 30 °C min^{-1} to 280 °C for 0.5 min.
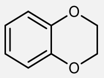	2-Hydroxy-benz-1.4-dioxan (**12a**)<br>9.76	80 °C holding for 2.0 min; 20 °C min^{-1} to 220 °C holding for 2.0 min; 30 °C min^{-1} to 280 °C for 0.5 min.
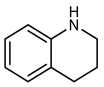	3,4-dihydro-2(1H)-quinolone (**17b**)<br>12.03	80 °C holding for 1.5 min; 20 °C min^{-1} to 220 °C holding for 2.0 min; 30 °C min^{-1} to 280 °C for 0.5 min.
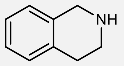	3,4-dihydroisoquinolin-1(2H)-one (**18b**)<br>12.69	80 °C holding for 1.5 min; 20 °C min^{-1} to 220 °C holding for 2.0 min; 30 °C min^{-1} to 280 °C for 0.5 min.

Column: SH-Rtx-1 (30 m × 0.25 mm × 0.25 µm).

3.5 Supplementary Table 5. Details of the HPLC chromatograph

Substrate	Column	Retention time [min]	Program
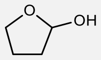	OJ-H (Chiralcel)	tetrahydrofuran-2-ol (***R***)<br>8.651<br>tetrahydrofuran-2-ol (***S***)<br>7.368	5% i-PrOH in hexane,<br>1.0 mL min^{-1},<br>30 ^{o}C, 230 nm
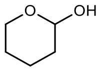	OJ-H (Chiralcel)	tetrahydropyran-2-ol (***R***)<br>6.632<br>tetrahydropyran-2-ol (***S***)<br>5.221	20% i-PrOH in hexane,<br>1.0 mL min^{-1},<br>30 ^{o}C, 220 nm
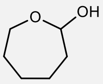	OJ-H (Chiralcel)	oxepan-2-ol (***R***)<br>8.831<br>oxepan-2-ol (***S***)<br>7.508	5% i-PrOH in hexane,<br>1.0 mL min^{-1},<br>30 ^{o}C, 230 nm
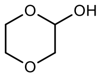	OJ-H (Chiralcel)	1,4-Dioxan-2-ol (***R***)<br>10.621<br>1,4-Dioxan-2-ol (***S***)<br>5.600	2% i-PrOH in hexane,<br>1.0 mL min^{-1},<br>30 ^{o}C, 220 nm
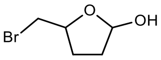	i-Amylose-1 (Phenomenex)	5-bromomethyl-tetrahydro-furan-2-ol (***R***)<br>7.847<br>5-bromomethyl-tetrahydro-furan-2-ol (***S***)<br>6.774	5% i-PrOH in hexane,<br>1.0 mL min^{-1},<br>30 ^{o}C, 220 nm
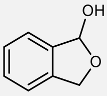	OJ-H (Chiralcel)	1,3-dihydroisobenzofuran-1-ol (***R***)<br>11.322<br>1,3-dihydroisobenzofuran-1-ol (***S***)<br>10.841	5% i-PrOH in hexane,<br>1.0 mL min^{-1},<br>30 ^{o}C, 230 nm
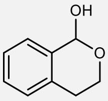	OJ-H (Chiralcel)	isochroman-1-ol (***R***)<br>10.73<br>isochroman-1-ol (***S***)<br>11.56	5% i-PrOH in hexane,<br>1.0 mL min^{-1},<br>30 ^{o}C, 230 nm
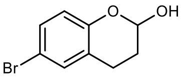	i-Amylose-1<br>(Phenomenex)	6-bromo-3,4-dihydro-2H-1-<br>benzopyran-2-ol (***R***)<br>9.972<br>6-bromo-3,4-dihydro-2H-1-	10% i-PrOH in hexane,<br>1.0 mL min^{-1},<br>30 ^{o}C, 220 nm

		benzopyran-2-ol (***S***)<br><br>7.995	
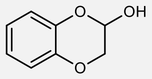	OJ-H (Chiralcel)	2-Hydroxy-benz-1.4-dioxan (***R***)<br><br>13.734<br><br>2-Hydroxy-benz-1.4-dioxan (***S***)<br><br>12.801	5% i-PrOH in hexane,<br><br>1.0 mL min^{-1},<br><br>30 ^{o}C, 254 nm

4. Supplementary References

1. Chen, S., Hong, B. C., Su, C. F., Sarshar, S. An unexpected inversion of enantioselectivity in the proline catalyzed intramolecular Baylis–Hillman reaction. *Tetrahedron Lett.* **46**, 8899-8903 (2005).
2. Gribble, A. D. *et al.* ATP-citrate lyase as a target for hypolipidemic intervention. 2. synthesis and evaluation of (3R*,5S*)-ω-substituted-3-carboxy-3,5-dihydroxyalkanoic acids and their γ-lactone prodrugs as inhibitors of the enzyme in vitro and in vivo. *J. Med. Chem.* **41**, 3582-3595 (1998).
3. Zuo, Y. J., Chang, X. T., Hao, Z. M., Zhong, C. M. Copper-catalyzed, stereoconvergent, cis-diastereoselective borylative cyclization of ω-mesylate-α,β-unsaturated esters and ketones. *Org. Biomol. Chem.* **15**, 6323-6327 (2017).
4. López-López, J. A. Guerra, F. M. Javier, Moreno-Dorado, F., Jorge, Z. D., Massanet, G. M. Synthesis of chlorinated β- and γ-lactones from unsaturated acids with sodium hypochlorite and Lewis acids. *Tetrahedron Lett.* **48**, 1749-1752 (2007).
5. Nguyen-Ba, P., Turcotte, N., Yuen, L., Bédard, J., Quimpère, M., Chan, L. Identification of novel nucleotide phosphonate analogs with potent anti-HCMV activity. *Bioorganic Med. Chem. Lett.* **8**, 3561-3566 (1998).
6. Chen, F., Jiang, X., Er, J. C., Yeung, Y. Y. Molecular sieves as an efficient and recyclable catalyst for bromolactonization and bromoacetoxylation reactions. *Tetrahedron Lett.* **51**, 3433-3435 (2010).
7. Mikami, K., Ohmura, H. Palladium-catalyzed isobenzofuran generation under neutral conditions via oxidative addition to lactol methyl ether. *Org. Lett.* **4**, 3355-3357 (2002).
8. Lee, S., Kaib, P. S. J., List, B. Asymmetric catalysis via cyclic, aliphatic oxocarbenium ions. *J. Am. Chem. Soc.* **139**, 2156-2159 (2017).
9. Enholm, E.J., Cottone, J. S., Allais, F. Highly diastereoselective 5-hexenyl radical cyclizations with Lewis acids and carbohydrate scaffolds. *Org. Lett.* **3**, 145-147 (2001).
10. Chen, Y., Li, G. X., Peng, A. Q., Tang, Y., Wang, L. Rapid construction of enantioenriched benzofurochromanes by SaBOX/Copper(II) catalyzed enantioselective [3 + 2] annulation of γ-chromenes with quinones. *Org. Lett.* **24**, 5525-5529 (2022).
11. Gandolfi, C. A. *et al.* N-Acyl-2-substituted-1,3-thiazolidines, a new class of non-narcotic antitussive agents: studies leading to the discovery of ethyl 2-[(2-methoxyphenoxy)methyl]-.beta.-oxothiazolidine-3-propanoate. *J. Med. Chem.* **38**, 508-525 (1995).
12. Molina-Espeja, P., Ma, S., Mate, D. M., Ludwig, R., Alcalde, M. Tandem-yeast expression system for engineering and producing unspecific peroxygenase. *Enzyme Microb. Technol.* **73–74**, 29-33 (2015).
13. Weckbecker, A., Hummel, W. Cloning, expression, and characterization of an (R)-specific alcohol dehydrogenase from Lactobacillus kefir. *Biocatal. Biotransformation* **24**, 380-389 (2006).
14. Ramirez-Escudero, M., Molina-Espeja, P., Gomez de Santos, P., Hofrichter, M., Sanz-Aparicio, J., Alcalde, M. Structural insights into the substrate promiscuity of a laboratory-evolved peroxygenase. *ACS Chem. Biol.* **13**, 3259-3268 (2018).
15. Søndergaard, C. R., Olsson, M. H. M., Rostkowski, M., Jensen, J. H. Improved treatment of

ligands and coupling effects in empirical calculation and rationalization of pKa values. *J. Chem. Theory Comput.* **7**, 2284-2295 (2011).

16. D.A. Case KB, I.Y. Ben-Shalom, S.R. Brozell, D.S. Cerutti, T.E. Cheatham, III, V.W.D. Cruzeiro, T.A. Darden, R.E. Duke, G. Giambasu, M.K. Gilson, H. Gohlke, A.W. Goetz,R Harris, S. Izadi, S.A. Iz_mailov, K. Kasavajhala, A. Kovalenko, R. Krasny, T.Kurtzman, T.S. Lee, S. LeGrand, P. Li, C. Lin, J. Liu, T. Luchko, R. Luo, V. Man, K.M. Merz, Y. Miao, O. Mikhailovskii, G. Monard, H. Nguyen, A. Onufriev, F. Pan, S. Pantano, R. Qi, D.R. Roe, A. Roitberg, C. Sagui, S. Schott-Verdugo, J. Shen, C.L. Simmerling, N.R. Skrynnikov, J. Smith, J. Swails, R.C. Walker, J. Wang, L. Wilson, R.M. Wolf, X. Wu, Y. Xiong, Y. Xue, D.M. York and P.A. Kollman. AMBER 2020, University of California, San Francisco. (2020).
17. Li, P., Merz, K. M., Jr. MCPB.py: A python based metal center parameter builder. *J. Chem. Inf. Model.* **56**, 599-604 (2016).
18. Shahrokh, K., Orendt, A., Yost, G. S., Cheatham III, T. E. Quantum mechanically derived AMBER-compatible heme parameters for various states of the cytochrome P450 catalytic cycle. *J. Comput. Chem.* **33**, 119-133 (2012).
19. Wang, J., Wolf, R. M., Caldwell, J. W., Kollman, P. A., Case, D. A. Development and testing of a general amber force field. *J. Comput. Chem.* **25**, 1157-1174 (2004).
20. Bayly, C. I., Cieplak, P., Cornell, W., Kollman, P. A. A well-behaved electrostatic potential based method using charge restraints for deriving atomic charges: the RESP model. *J. Chem. Phys.* **97**, 10269-10280 (1993).
21. Gaussian 16 RC, M. J. Frisch, G. W. Trucks, H. B. Schlegel, G. E. Scuseria, M. A. Robb, J. R. Cheeseman, G. Scalmani, V. Barone, G. A. Petersson, H. Nakatsuji, X. Li, M. Caricato, A. V. Marenich, J. Bloino, B. G. Janesko, R. Gomperts, B. Mennucci, H. P. Hratchian, J. V. Ortiz, A. F. Izmaylov, J. L. Sonnenberg, D. Williams-Young, F. Ding, F. Lipparini, F. Egidi, J. Goings, B. Peng, A. Petrone, T. Henderson, D. Ranasinghe, V. G. Zakrzewski, J. Gao, N. Rega, G. Zheng, W. Liang, M. Hada, M. Ehara, K. Toyota, R. Fukuda, J. Hasegawa, M. Ishida, T. Nakajima, Y. Honda, O. Kitao, H. Nakai, T. Vreven, K. Throssell, J. A. Montgomery, Jr., J. E. Peralta, F. Ogliaro, M. J. Bearpark, J. J. Heyd, E. N. Brothers, K. N. Kudin, V. N. Staroverov, T. A. Keith, R. Kobayashi, J. Normand, K. Raghavachari, A. P. Rendell, J. C. Burant, S. S. Iyengar, J. Tomasi, M. Cossi, J. M. Millam, M. Klene, C. Adamo, R. Cammi, J. W. Ochterski, R. L. Martin, K. Morokuma, O. Farkas, J. B. Foresman, D. J. Fox, Gaussian. Inc., Wallingford CT. (2016).
22. Tian, C. *et al.* ff19SB: Amino-acid-specific protein backbone parameters trained against quantum mechanics energy surfaces in solution. *J. Chem. Theory Comput.* **16**, 528-552 (2020).
23. Jorgensen, W., Chandrasekhar, J., Madura, J., Impey, R., Klein, M. Comparison of simple potential functions for simulating liquid water. *J. Chem. Phys.* **79**, 926-935 (1983).
24. Lee, C., Yang, W., Parr, R. G. Development of the Colle-Salvetti correlation-energy formula into a functional of the electron density. *Phys. Rev. B* **37**, 785-789 (1988).
25. Becke, A. D. Density-functional thermochemistry. III. The role of exact exchange. *J. Chem. Phys.* **98**, 5648-5652 (1993).
26. Grimme, S. Density functional theory with London dispersion corrections. *WIREs Comput. Mol. Sci.* **1**, 211-228 (2011).
27. Bursch, M., Caldeweyher, E., Hansen, A., Neugebauer, H., Ehlert, S., Grimme, S. Understanding and quantifying london dispersion effects in organometallic complexes. *Acc. Chem. Res.* **52**,

258-266 (2019).

28. Shaik, S., Kumar, D., de Visser, S. P., Altun, A., Thiel, W. Theoretical perspective on the dtructure and mechanism of cytochrome P450 enzymes. *Chem. Rev.* **105**, 2279-2328 (2005).

29. Shaik, S., Cohen, S., Wang, Y., Chen, H., Kumar, D., Thiel, W. P450 enzymes: their structure, reactivity, and selectivity—modeled by QM/MM calculations. *Chem. Rev.* **110**, 949-1017 (2010).

30. Siegbahn, P. E. M., Blomberg, M. R. A. A systematic DFT approach for studying mechanisms of redox active enzymes. *Front. Chem.* **6** (2018).

31. Siegbahn, P. E. M. A quantum chemical approach for the mechanisms of redox-active metalloenzymes. *RSC Adv.* **11**, 3495-3508 (2021).

32. Weigend, F., Ahlrichs, R. Balanced basis sets of split valence, triple zeta valence and quadruple zeta valence quality for H to Rn: Design and assessment of accuracy. *Phys. Chem. Chem. Phys.* **7**, 3297-3305 (2005).

33. Marenich, A. V., Cramer, C. J., Truhlar, D. G. Universal solvation model based on solute electron density and on a continuum model of the solvent defined by the bulk dielectric constant and atomic surface tensions. *J. Phys. Chem. B* **113**, 6378-6396 (2009).

34. Torrie, G. M., Valleau, J. P. Nonphysical sampling distributions in Monte Carlo free-energy estimation: Umbrella sampling. *J. Comput. Phys.* **23**, 187-199 (1977).

35. Isralewitz, B., Gao, M., Schulten, K. Steered molecular dynamics and mechanical functions of proteins. *Curr. Opin. Struct. Biol.* **11**, 224-230 (2001).

36. Kumar, S., Rosenberg, J. M., Bouzida, D., Swendsen, R. H., Kollman, P. A. THE weighted histogram analysis method for free-energy calculations on biomolecules. I. The method. *J. Comput. Chem.* **13**, 1011-1021 (1992).
