# Supplementary material for: Reaction engineering blocks ether cleavage for synthesizing chiral cyclic hemiacetals catalyzed by unspecific peroxygenase
Source: Nat Commun. 2024 Feb 9;15:1235. doi: 10.1038/s41467-024-45545-z (PMC10858125; doi:10.1038/s41467-024-45545-z)
Supplement: Supplementary file 3 — Reporting Summary [file 41467_2024_45545_MOESM3_ESM.pdf]

nature portfolio

Corresponding author(s): Peigao Duan, Xiang Sheng, Wuyuan Zhang

Last updated by author(s): Dec 17, 2023

Reporting Summary

Nature Portfolio wishes to improve the reproducibility of the work that we publish. This form provides structure for consistency and transparency in reporting. For further information on Nature Portfolio policies, see our Editorial Policies and the Editorial Policy Checklist.

Statistics

For all statistical analyses, confirm that the following items are present in the figure legend, table legend, main text, or Methods section.

n/a	Confirmed	
☐	☒	The exact sample size (*n*) for each experimental group/condition, given as a discrete number and unit of measurement
☐	☒	A statement on whether measurements were taken from distinct samples or whether the same sample was measured repeatedly
☒	☐	The statistical test(s) used AND whether they are one- or two-sided<br>*Only common tests should be described solely by name; describe more complex techniques in the Methods section.*
☒	☐	A description of all covariates tested
☒	☐	A description of any assumptions or corrections, such as tests of normality and adjustment for multiple comparisons
☐	☒	A full description of the statistical parameters including central tendency (e.g. means) or other basic estimates (e.g. regression coefficient) AND variation (e.g. standard deviation) or associated estimates of uncertainty (e.g. confidence intervals)
☒	☐	For null hypothesis testing, the test statistic (e.g. *F*, *t*, *r*) with confidence intervals, effect sizes, degrees of freedom and *P* value noted<br>*Give P values as exact values whenever suitable.*
☒	☐	For Bayesian analysis, information on the choice of priors and Markov chain Monte Carlo settings
☒	☐	For hierarchical and complex designs, identification of the appropriate level for tests and full reporting of outcomes
☒	☐	Estimates of effect sizes (e.g. Cohen's *d*, Pearson's *r*), indicating how they were calculated

Our web collection on statistics for biologists contains articles on many of the points above.

Software and code

Policy information about availability of computer code

Data collection	No novel software or code was established in this work. Gas Chromatography (GC) was recorded on a GC2010 Pro from SHIMADZU. High Performance Liquid Chromatography (HPLC) was recorded on a LC-2010C HT instrument from SHIMADZU. GaussView 6.0 was used to prepare the ligand structure. Molecular dynamics (MD) simulations, steered MD and umbrella sampling (UM) were performed using the software Amber20 (non-commercial version). Quantum chemical (QM) calculations were conducted using the commercial software Gaussian16.C.
Data analysis	GC and HPLC data were analyzed using the software LabSolutions™(version 5.90 LE). 1H- and 13C-NMR spectra were recorded on Bruker (400 MHz and 101 MHz, respectively). The MD and steered MD data were analyzed using Amber20 (non-commercial version), while the UM data was processed using Amber20 (non-commercial version) and the open-source software WHAM. The QM calculations data was analyzed using GaussView 6.0.

For manuscripts utilizing custom algorithms or software that are central to the research but not yet described in published literature, software must be made available to editors and reviewers. We strongly encourage code deposition in a community repository (e.g. GitHub). See the Nature Portfolio guidelines for submitting code & software for further information.

Data

Policy information about availability of data

All manuscripts must include a data availability statement. This statement should provide the following information, where applicable:

- Accession codes, unique identifiers, or web links for publicly available datasets
- A description of any restrictions on data availability
- For clinical datasets or third party data, please ensure that the statement adheres to our policy

All data generated and analyzed in this study can be found in the article and its Supplementary Information. The source data underlying Table 1, Figures 1a-b, Figures 3a-b, Figures 6a-b, and Supplementary Figures 2–5 are provided as a source data file. PDB file used in this study is available in Protein Data Bank (PDB) (ID: 6ekz, https://doi.org/10.2210/pdb6ekz/pdb), the sequence of Lactobacillus kefiri data is available in National Center for Biotechnology Information (NCBI) (GenBank: AY267012.1, https://www.ncbi.nlm.nih.gov/gene/71566997/).These data are also available from the corresponding authors upon request. Source data are provided with this paper.

Research involving human participants, their data, or biological material

Policy information about studies with human participants or human data. See also policy information about sex, gender (identity/presentation), and sexual orientation and race, ethnicity and racism.

Reporting on sex and gender	n/a
Reporting on race, ethnicity, or other socially relevant groupings	n/a
Population characteristics	n/a
Recruitment	n/a
Ethics oversight	n/a

Note that full information on the approval of the study protocol must also be provided in the manuscript.

Field-specific reporting

Please select the one below that is the best fit for your research. If you are not sure, read the appropriate sections before making your selection.

☒ Life sciences ☐ Behavioural & social sciences ☐ Ecological, evolutionary & environmental sciences

For a reference copy of the document with all sections, see nature.com/documents/nr-reporting-summary-flat.pdf

Life sciences study design

All studies must disclose on these points even when the disclosure is negative.

Sample size	No sample size calculations were performed for this study. Sample sizes are indicated for each experiment and were chosen based on similar studies. Experiments were performed in duplicate.
Data exclusions	No data was excluded.
Replication	All attempts at replication were successful. We generally performed duplicate independent experiments.
Randomization	n/a, no claims on randomization, this study does not involve animal or human subjects or group allocation.
Blinding	n/a, no claims on blinding, this study does not involve animal or human subjects or group allocation.

Reporting for specific materials, systems and methods

We require information from authors about some types of materials, experimental systems and methods used in many studies. Here, indicate whether each material, system or method listed is relevant to your study. If you are not sure if a list item applies to your research, read the appropriate section before selecting a response.

Materials & experimental systems

n/a	Involved in the study
x	Antibodies
x	Eukaryotic cell lines
x	Palaeontology and archaeology
x	Animals and other organisms
x	Clinical data
x	Dual use research of concern
x	Plants

Methods

n/a	Involved in the study
x	ChIP-seq
x	Flow cytometry
x	MRI-based neuroimaging

Plants

Seed stocks	n/a
Novel plant genotypes	n/a
Authentication	n/a
